# Comparing Disease‐Free Survival (DFS) and Overall Survival (OS) Rates in Breast Cancer Patients: Axillary Lymph Node Dissection (ALND) Versus Sentinel Lymph Node Biopsy (SLNB)

**DOI:** 10.1155/ijbc/5039446

**Published:** 2026-06-26

**Authors:** Bahar Amiri, Mahta Mellat Ardakani, Ali Pajouhi, Atieh Akbari, Simin Farokhi, Mohammad Esmaeil Akbari, Samaneh Tahmasebi Ghorabi, Arian Karimi Rouzbahani, Mania Beiranvand

**Affiliations:** ^1^ Student Research Committee, Faculty of Medicine, Lorestan University of Medical Sciences, Khorramabad, Iran, lums.ac.ir; ^2^ Razi Herbal Medicines Research Center, Lorestan University of Medical Sciences, Khorramabad, Iran, lums.ac.ir; ^3^ USERN Office, Lorestan University of Medical Sciences, Khorramabad, Iran, lums.ac.ir; ^4^ Cancer Research Center, Shahid Beheshti University of Medical Sciences, Tehran, Iran, sbmu.ac.ir; ^5^ Surgery Education and Research Network (SERGN), Universal Scientific Education and Research Network (USERN), Lorestan University of Medical Sciences, Melbourne, Australia, lums.ac.ir; ^6^ Maxillofacial Surgery and Implantology and Biomaterial Research Foundation, Khorramabad, Iran; ^7^ Department of General Surgery, Faculty of Medicine, Lorestan University of Medical Sciences, Khorramabad, Iran, lums.ac.ir

**Keywords:** ALND, breast cancer, disease-free survival, overall survival, SLNB

## Abstract

**Background:**

Examining the survival rate in breast cancer patients serves as a metric for assessing and advancing various treatment modalities. In light of the significant incidence of breast cancer in Iran, this study is aimed at examining and comparing the disease‐free survival (DFS) and overall survival (OS) rates among breast cancer patients who underwent axillary lymph node dissection (ALND) and sentinel lymph node biopsy (SLNB).

**Patients and Methods:**

This retrospective cohort research included 1071 patients at Tajrish Hospital, Shahid Beheshti University of Medical Sciences, Tehran, Iran, from 1991 to 2021. The recurrence and survival rates of patients undergoing ALND and SLNB in both groups were examined and evaluated. The data were analyzed using SPSS statistical software using descriptive statistical techniques (percentage, frequency, mean, and standard deviation), Kaplan–Meier analysis, and Cox regression tests. A significance threshold of 0.05 is used.

**Results:**

The average OS among all patients was 18.08 ± 0.51. The average OS in the ALND group was 18.1 ± 0.53; in the SLNB group, it was 12.8 ± 0.39, which was insignificant (*p* < 0.05). The average DFS among all patients was 17.13 ± 0.51. The average DFS in the ALND group was 16.95 ± 0.54; in the SLNB group, it was 11.96 ± 0.3; the difference was insignificant (*p* = 0.11).

**Conclusion:**

OS and DFS in the ALND group were higher than in the SLNB group, but they were not statistically significant.

## 1. Introduction

In 2020, female breast cancer is expected to account for 2.3 million new cases, or 11.7% of all cancer cases, surpassing lung cancer as the main cause of cancer incidence worldwide. It accounts for 685,000 deaths globally, making it the sixth most common cause of cancer mortality (1). There are numerous risk factors such as sex, aging, estrogen, early onset of menstruation (12 years) and late termination (50 years) (2), family history, BRCA1 and BRCA2 gene mutations, hormone replacement therapy, obesity, alcohol consumption, lack of physical activity, or improper diet, which can increase the possibility of developing breast cancer (2–6). Invariably, breast cancer develops in silence. The majority of people learn about their illness during regular checkups. Early detection increases the chances of survival (7). To diagnose breast cancer, a physical examination and imaging, including mammography and tissue biopsy, should be performed. In the treatment of breast cancer, surgery is crucial (8). With improved quality of life and comparable or noninferior survival results, conservative surgery has replaced conventional radical surgery as the surgical treatment of breast cancer for the last 50 years. Sentinel lymph node biopsy (SLNB) has replaced axillary lymph node dissection (ALND) for axillary staging, and breast‐conserving surgery (BCS) has replaced radical mastectomy for the majority of patients (9).

Cancer cells are more likely to move from the main tumor to the first lymph node, which is known as a sentinel lymph node (SLN). For patients with clinically node‐negative early‐stage breast cancer, SLNB is a technique. The SLNs are located, extracted, and analyzed using this technique to detect the presence or absence of cancer cells in the lymph nodes. With this information, the physician may establish a suitable treatment plan and ascertain the cancer’s stage or the extent of the illness in the body (3). For patients with clinically node‐positive breast cancer, ALND is the recommended first course of treatment (10).

Investigating whether ALND and SLNB are linked to improved prognoses and longer disease‐free survival (DFS) and overall survival (OS) rates in patients with breast cancer is the aim of this research. Even while the creation of novel medicines for breast cancer has advanced significantly, there is currently insufficient data to substantiate the idea that these drugs improve patient survival. Recent therapies, however, have been shown to improve survival rates in some investigations.

Given the importance of breast cancer and its high incidence, we chose to compare the OS and DFS rates of breast cancer patients treated with SLNB and ALND at the Cancer Research Center of Shahid Beheshti University of Medical Sciences between 1991 and 2021.

## 2. Methods

### 2.1. Study Design and Participants

This retrospective cohort research was conducted at Tajrish Hospital, Shahid Beheshti University of Medical Sciences, Tehran, Iran, from 1991 to 2021. The study procedure was reviewed and sanctioned by the Ethics Committee of the Cancer Study Center at Shahid Beheshti University in Tehran. All patients provided written, informed, and voluntary consent. The checklists were created anonymously, ensuring the confidentiality of patients’ personal information. The tenets of the Helsinki Declaration were adhered to.

This research analyzed the medical records of breast cancer patients who had ALND and SLNB from 1991 to 2021. Only patients monitored for at least 6 months posttreatment to assess recurrence and calculate survival were included.

The inclusion criteria encompassed hospitalization for breast cancer with subsequent ALND or SLNB at the Shahid Beheshti University of Medical Sciences Cancer Research Center, breast cancer patients monitored for a minimum of 6 months posttreatment, accessibility to clinical records from 1991 to 2021, and patient satisfaction. Patients were omitted if they had insufficient medical records, were reluctant to maintain participation, or failed to adhere to follow‐up therapy.

The sampling technique used was census sampling. A total of 1071 patients who satisfied the inclusion criteria were included.

Patients are categorized into two groups: the case group, consisting of individuals with ALND from the outset, and the control group, including those who have undergone SLNB for the first time. If the nodes in frozen blood are positive, a restricted dissection of four to five nodes is conducted.

Subgroup analyses not directly related to the primary study objective were considered exploratory and are presented in the Supporting Information to maintain focus on the primary comparison between ALND and SLNB.

ALND was conducted under recognized protocols. The axillary lymph nodes were evaluated using ultrasound and fine‐needle aspiration for a SLNB. Clinically negative nodes were defined as nodes that showed normal on ultrasonography or were confirmed negative by fine‐needle aspiration. The only tracer used for SLNB was 99mTc‐rituximab. Tracer injection was conducted at two designated locations: the breast parenchyma next to the tumor and the subcutaneous and subareolar tissues. An SLNB was performed under local anesthesia. The specimens were fixed in formalin and then embedded in paraffin. The specimens were subsequently sectioned at 5 mm intervals and subjected to staining with hematoxylin and eosin.

### 2.2. Follow‐Up and Outcomes

The data‐gathering instruments for this study included a researcher‐developed demographic questionnaire, patients’ clinical records, and pathology reports.

The examination of patients included the documentation of demographic factors such as age at diagnosis, marital status, employment, economic position, number of children, family history of breast cancer, family history of any cancer, and infertility. Clinical and pathological data of the examined patients, encompassing tumor size, tumor grade, quantity of masses, tumor type, tumor histology, status and count of positive lymph nodes in the axilla, hormone therapy, number of excised lymph nodes, count of positive lymph nodes, chemotherapy history, radiotherapy history, and cause of death, was gathered and documented in the checklist.

Patients are assessed based on clinical and pathological characteristics. The response variables were delineated as DFS (the duration from the date of breast surgery to the date of the last consultation or recurrence) and OS from the date of surgery to the date of death. The duration of the patient′s participation in the study (from admission to the local recurrence of breast cancer) was evaluated, along with other variables including age, number of offspring, history of infertility, marital status, family history (in first‐ or second‐degree relatives), tumor grade, tumor size, and glandular involvement. Lymphatic status, surgical intervention, adjuvant therapy, the quantity of positive lymph nodes, and tumor histology are independent factors. The recurrence and survival rates of patients undergoing ALND and SLNB in both cohorts were examined and contrasted.

DFS was defined as the time from the date of primary breast surgery to the date of the first documented event, including locoregional recurrence, distant metastasis, contralateral breast cancer, or death from any cause, whichever occurred first. Patients without an event were censored at the date of last follow‐up.

OS was defined as the time from the date of surgery to death from any cause or last follow‐up.

### 2.3. Data Analysis

The collected data were analyzed using SPSS software Version 22. Measures of central tendency and index of dispersion were calculated. The data were analyzed using descriptive statistical methods (percentage, frequency, mean, and standard deviation), Kaplan–Meier analysis, and Cox regression tests. Median survival times were calculated using Kaplan–Meier analysis. A significant level of 0.05 is considered. Due to retrospective data limitations and available statistical resources, multivariable adjustment and propensity score matching were not performed.

## 3. Results

In this retrospective cohort study, which was conducted to compare OS and DFS in breast cancer patients who underwent ALND and SLNB in the Tajrish Hospital of Shahid Beheshti University of Medical Sciences from 1991 to 2021, the data of 1074 patients from the Cancer Research Center of Shahid Beheshti University of Medical Sciences was received.

### 3.1. Comparison of the OS Rate Between the ALND and SLNB Groups

**Figure 1:**
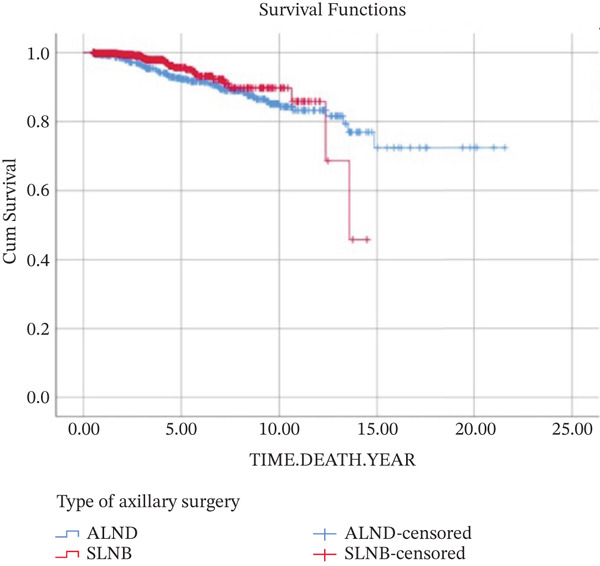
Comparison of the overall survival rate between the ALND and SLNB groups (*p* = 0.25).

During this study, after receiving the patients’ data file from the Shahid Beheshti University of Tehran, it was determined that 1074 people had undergone SLNB and ALND during the study period, of which 505 people were in the ALND group and 566 people were in the SLNB group. Of these, 75 deaths were recorded, 52 of whom underwent ALND and 23 of whom underwent SLNB. The average OS among all patients was 18.08 ± 0.51. The average OS in the ALND group was 18.1 ± 0.53; in the SLNB group, it was 12.8 ± 0.39, which was not statistically significant (*p* = 0.25) (Table 1 and Figure 1).

**Table 1:** Comparison of the overall survival rate between the ALND and SLNB groups (*p* = 0.25).

Type of axillary surgery	Average	Standard deviation	95% confidence interval	
			Lower bound	Upper bound
ALND	18.105	0.532	17.061	19.148
SLNB	12.806	0.399	12.024	13.587
All patients	18.087	0.512	17.083	19.090

The mean OS of the entire cohort was numerically similar to that of the ALND group. This reflects the imbalance in survival time distribution between the two groups and the unequal contribution of censored observations. Because mean survival estimates in the Kaplan–Meier analysis are influenced by censoring patterns and follow‐up duration, the overall cohort mean does not represent a simple weighted average of subgroup means. Furthermore, patients undergoing ALND were more likely to be treated in earlier treatment eras with longer follow‐up duration, which contributed to a higher cumulative survival estimate in this group. These factors may explain the apparent similarity between overall cohort survival and ALND survival.

### 3.2. Comparison of the DFS Rate Between the ALND and SLNB Groups

**Figure 2:**
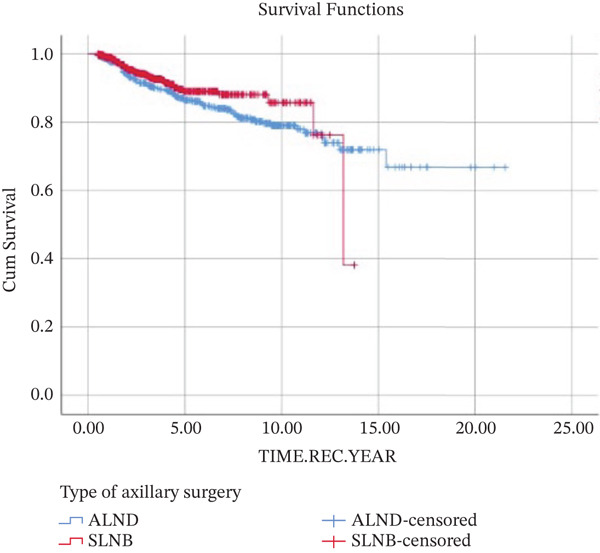
Comparison of disease‐free survival rate between the ALND and SLNB groups (*p* = 0.11).

During this study, after receiving the patient data file from the Shahid Beheshti University of Tehran, it was determined that 1074 people had undergone SLNB and ALND during the study period, of which 507 people were in the ALND group and 567 people were in the SLNB group. Of these, 123 events (recurrence, metastasis, and death) were recorded, of which 79 underwent ALND and 44 underwent SLNB. The average DFS among all patients was 17.13 ± 0.51. The average DFS in the ALND group was 16.95 ± 0.54; in the SLNB group, it was 11.96 ± 0.3, which was not statistically significant (*p* = 0.11) (Table 2 and Figure 2).

**Table 2:** Comparison of disease‐free survival rate between the ALND and SLNB groups (*p* = 0.11).

Type of axillary surgery	Average	Standard deviation	95% confidence interval	
			Lower bound	Upper bound
ALND	16.950	0.547	15.877	18.023
SLNB	11.960	0.306	11.360	12.559
All patients	17.134	0.513	16.128	18.140

### 3.3. The OS Rate According to Gender

The study examined the gender of the patients, 1066 of whom were women and 5 of whom were men. Of these, 73 deaths were recorded among women and two among men. The average OS in women was 18.2 ± 0.5 and in men 10.14 ± 2.83, which the difference was not statistically significant (*p* = 0.07) (Table S3 and Figure S3).

### 3.4. The Rate of DFS According to Gender

In examining the gender of the patients examined in the study, it was found that 1069 were women and 5 were men. Of these, 122 deaths were recorded among women and one incident (recurrence, metastasis, and death) among men. The average DFS in women was 17.19 ± 0.5 and in men 11.29 ± 0.34, which was not statistically significant (*p* = 0.855) (Table S4 and Figure S4).

### 3.5. The OS Rate of the Studied Patients According to Family History

In examining the family history of the patients examined in the study, it was found that 686 people had no family history, 145 people had a family history in first‐degree relatives, and 137 people had a family history in second‐degree relatives. Forty‐eight cases of death among the patients who had no family history, 8 cases of death among the patients who had a family history in their first‐degree relatives, and 10 cases of death among the patients who had a family history in their second‐degree relatives were registered. The average OS in patients with no family history was equal to 16.63 ± 0.6; in patients with family history in first‐degree relatives, it was 14.41 ± 0.37; in patients with family history in second‐degree relatives, it was 18.39 ± 1.06, which was not statistically significant. (*p* = 0.63) (Table S5 and Figure S5).

### 3.6. The Rate of DFS in the Studied Patients According to Family History

In examining the family history of the patients examined in the study, it was found that 688 people had no family history, 146 people had a family history with first‐degree relatives, and 137 people had a family history with second‐degree relatives. Among the patients who had no family history, 85 cases occurred; among the patients who had a family history in first‐degree relatives, 12 cases; and among the patients who had a family history in second‐degree relatives, 17 cases (recurrence, metastasis, and death) were registered. The average DFS in patients with no family history was equal to 15.29 ± 0.58; in patients with a family history of first‐degree relatives, it was 13.76 ± 0.52, and in patients with a family history of second‐degree relatives, it was 18.12 ± 0.78, which is statistically significant. It was not significant (*p* = 0.26) (Table S6 and Figure S6).

### 3.7. The OS Rate of the Studied Patients According to the Type of Surgery

In the examination of the type of surgery of the patients investigated in the study, it was found that 893 people underwent BCS, 144 underwent modified radical mastectomy (MRM), and 12 underwent BCS/MRM. The type of surgery was unknown to 22 people. There were 42 deaths among patients who underwent BCS, 28 deaths among patients who underwent MRM, and four deaths among patients who underwent BCS/MRM. The average OS in patients under BCS was equal to 19.31 ± 0.45; in patients under MRM, it was equal to 15.73 ± 0.88, and in patients under BCS/MRM, it was equal to 11.54 ± 1.79, which the difference was statistically significant (*p* ≤ 0.001) (Table S7 and Figure S7).

### 3.8. The Rate of DFS in the Studied Patients According to the Type of Surgery

In the examination of the type of surgery of the patients examined in the study, it was found that 894 people underwent BCS, 146 underwent MRM, and 12 underwent BCS/MRM. The type of surgery was unknown to 22 people. Among the patients who underwent BCS, 73 cases were recorded; among the patients who underwent MRM, 32 cases were recorded; and among the patients who underwent BCS/MRM, 12 cases of events (recurrence, metastasis, and death) were recorded. The average DFS in patients under BCS was equal to 17.62 ± 0.73; in patients under MRM, it was equal to 15.57 ± 0.84, and in patients under BCS/MRM, it was equal to 2.9 ± 0.6, and this difference was statistically significant (*p* ≤ 0.001) (Table S8 and Figure S8).

### 3.9. The OS Rate of the Studied Patients According to Lymphatic Vascular Invasion

In the investigation of lymphatic vascular invasion in the patients examined in the study, it was found that 300 people had an invasion and 627 had no invasion, and lymphatic vascular invasion was unclear in 135 patients. Among the patients who had an invasion, 32 cases were recorded; among the patients who did not have a lymphatic vascular invasion, 26 cases were recorded; and among those whose status of lymphatic vascular invasion was unclear, 17 cases of death were recorded. It was also found that the average OS in months in patients with lymphatic vascular invasion was 13.21 ± 0.71; in patients without lymphatic vascular invasion, it was 19.65 ± 0.44; and in patients whose status of lymphatic vascular invasion was unclear, it was 16.19 ± 1.18, which is the difference. It was statistically significant (*p* ≤ 0.001) (Table S9 and Figure S9).

### 3.10. The Rate of DFS in the Studied Patients According to Lymphatic Vascular Invasion

In the investigation of lymphatic vascular invasion in the patients examined in the study, it was found that 301 people had an invasion and 629 people had no invasion, and lymphatic vascular invasion was unclear in 135 patients. Among the patients with invasion, 52 cases were recorded; among the patients without lymphatic vascular invasion, 48 cases were recorded; and among those whose lymphatic vascular invasion status was unclear, 23 events (recurrence, metastasis, and death) were recorded. It was also found that the average DFS in months in patients with lymphatic vascular invasion was 12.72 ± 0.64; in patients without lymphatic vascular invasion, it was 18.02 ± 0.71, and in patients whose lymphatic vascular invasion status was unclear, it was 12.72 ± 0.64. This difference was statistically significant (*p* ≤ 0.001) (Table S10 and Figure S10).

### 3.11. The OS Rate in the Studied Patients According to the Presence of Hormone Receptor

In examining the presence of ER hormone receptors in the patients examined in the study, it was found that 714 people had ER hormone receptors, 268 people did not have ER hormone receptors, and the presence of ER hormone receptors was unknown in 40 patients. Among the patients who had an ER hormone receptor, 42 cases were recorded; among the patients who did not have an ER hormone receptor, 26 cases were recorded; among those whose ER hormone receptor status was unknown, 6 cases of death were recorded. It was also found that the average OS time in months in patients with ER hormone receptor was 16.92 ± 0.61; in patients without hormone receptor, it was 18.43 ± 0.58, and in patients whose ER hormone receptor status was unclear, it was 10.40 ± 0.737, which is the difference. It was statistically significant (*p* = 0.007) (Table S11 and Figure S11).

### 3.12. The Rate of DFS in the Studied Patients According to the Presence of the ER Hormone Receptor

In examining the presence of ER hormone receptors in the patients examined in the study, it was found that 715 people had ER hormone receptors, 270 people did not have ER hormone receptors, and the presence of ER hormone receptors was unknown in 40 patients. Among the patients who had ER hormone receptor, 74 cases were recorded; among the patients who did not have ER hormone receptor, 44 cases were recorded; and among those whose ER hormone receptor status was unclear, two events (recurrence, metastasis, and death) were recorded. It was also found that the average OS time in months in patients with ER hormone receptor was 16.14 ± 0.65; in patients without hormone receptor, it was 16.37 ± 0.58, and in patients whose ER hormone receptor status was unclear, it was 11.77 ± 0.42. This difference was statistically significant (*p* = 0.009) (Table S12 and Figure S12).

### 3.13. The OS Rate in the Studied Patients According to the Presence of PR Hormone Receptor

In the examination of the presence of PR receptor hormone in the patients examined in the study, it was found that 668 people had PR hormone receptors and 316 people did not have PR hormone receptors. The presence of PR receptor hormone was unknown in 42 patients. Among the patients who had PR hormone receptors, 43 cases were recorded; among the patients who did not have PR hormone receptors, 25 cases were recorded; and among the people whose PR hormone status was unknown, 6 cases of death were recorded. It was also found that the average OS time in months in patients with PR hormone receptor was 16.79 ± 0.61; in patients without hormone receptor, it was 18.99 ± 0.5, and in patients whose PR hormone status was unclear, it was 10.51 ± 0.7, which is the difference. It was statistically significant (*p* = 0.05) (Table S13 and Figure S13).

### 3.14. The DFS Rate in the Studied Patients According to the Presence of PR Hormone Receptor

In examining the presence of PR receptor hormone in the patients studied in the study, it was found that 668 people had PR hormone receptors and 319 people did not have PR hormone receptors. The presence of PR receptor hormone was unknown in 42 patients. Among the patients who had PR hormone receptor, 65 cases were recorded; among the patients who did not have PR hormone receptor, 54 cases were recorded; and among the people whose PR hormone receptor status was unknown, two events (recurrence, metastasis, and death) were recorded. It was also found that the average OS time in months in patients with PR hormone receptor was 16.25 ± 0.66; in patients without receptor hormone, it was 16.07 ± 0.8, and in patients whose PR hormone receptor status was unclear, it was 11.8 ± 0.39, which is the difference. It was statistically significant (*p* = 0.001) (Table S14 and Figure S14).

### 3.15. The OS Rate in the Studied Patients According to the Presence of the P53 Gene

In examining the presence of the P53 gene in the patients studied in the study, it was found that 668 people had the P53 gene and 316 people did not have the P53 gene, and the presence of the P53 gene was unknown in 42 patients. Among the patients who had the P53 gene, 43 cases were recorded; among the patients who did not have the P53 gene, 25 cases were recorded; and among those whose P53 gene status was unknown, 6 cases of death were recorded. It was also found that the average OS time in months in patients with the P53 gene was 16.79 ± 0.61; in patients without the P53 gene, it was 18.99 ± 0.5; and in patients whose P53 gene status was unclear, it was 10.51 ± 0.7. It was not statistically significant (*p* = 0.168) (Table S15 and Figure S15).

### 3.16. The Rate of DFS in the Studied Patients According to the Presence of the P53 Gene

In examining the presence of the P53 gene in the patients studied, it was found that 668 people had the P53 gene, 316 people did not have the P53 gene, and the presence of the P53 gene was unknown in 42 patients. Among the patients who had the P53 gene, 43 cases were recorded; among the patients who did not have the P53 gene, 25 cases were recorded; and among those whose P53 gene status was unknown, 6 cases of events (recurrence, metastasis, and death) were recorded. It was also found that the average OS time in months in patients with the P53 gene was 16.79 ± 0.61; in patients without the P53 gene, it was 14.85 ± 0.5; and in patients whose P53 gene status was unclear, it was 10.51 ± 0.7. It was not statistically significant (*p* = 0.846) (Table S16 and Figure S16).

### 3.17. The OS Rate of the Studied Patients According to the Presence of the HER‐2 Gene

In examining the presence of the HER‐2 gene in the patients examined in the study, it was found that 207 people had the HER‐2 gene, 113 people did not have the HER‐2 gene, and the presence of the HER‐2 gene was unknown in 751 patients. Among the patients with the HER‐2 gene, 19 cases were recorded; among the patients who did not have the HER‐2 gene, 7 cases were recorded; and among those whose HER‐2 gene status was unknown, 49 cases of death were recorded. It was also found that the average OS in patients with the HER‐2 gene was 17.17 ± 0.57; in patients without the gene, it was 17.13 ± 1.24, which was not statistically significant (*p* = 0.536) (Table S17 and Figure S17).

### 3.18. The Rate of DFS in the Studied Patients According to the Presence of the HER‐2 Gene

In examining the presence of the HER‐2 gene in the studied patients, it was found that 208 people had the HER‐2 gene and 114 people did not have the HER‐2 gene, and the presence of the HER‐2 gene was unknown in 752 patients. Among the patients who had the HER‐2 gene, 36 cases were recorded; among the patients who did not have the HER‐2 gene, 14 cases were recorded; and among those whose HER‐2 gene status was unknown, 73 cases of events (recurrence, metastasis, and death) were recorded. It was also found that the average DFS in patients with the HER‐2 gene was 15.32 ± 0.75; in patients without the gene, it was 13.08 ± 0.63, and this difference was statistically significant (*p* = 0.02) (Table S18 and Figure S18).

### 3.19. The OS Rate of the Studied Patients According to Chemotherapy

In the chemotherapy examination of the studied patients, it was found that 740 people had chemotherapy, 79 people had neoadjuvant chemotherapy, and 158 people had no chemotherapy. The chemotherapy status of 90 patients was not known. There were 62 deaths among people who had chemotherapy, 8 cases among people who had neoadjuvant, 4 cases among people whose chemotherapy was unclear, and 1 death among people who did not have chemotherapy. The average OS in patients with chemotherapy was 17.98 ± 0.54; in patients with neoadjuvant chemotherapy, it was 11.33 ± 1.03, and in patients without chemotherapy, it was 12.28 ± 0.9, which was statistically significant (*p* = 0.002) (Table S19 and Figure S19).

### 3.20. The Rate of DFS in the Studied Patients According to Chemotherapy

In the chemotherapy examination of the studied patients, it was found that 743 people had chemotherapy, 79 people had neoadjuvant chemotherapy, and 158 people had no chemotherapy. The chemotherapy status of 90 patients was not known. There were 98 cases among people who had chemotherapy, 8 cases among people who had neoadjuvant, 8 cases among people whose chemotherapy was unclear, and 9 cases of events (recurrence, metastasis, and death) among people who did not have chemotherapy. The average DFS in patients with chemotherapy was 16.99 ± 0.55; in patients with neoadjuvant chemotherapy, it was 12.82 ± 1.09, and in patients without chemotherapy, it was 11.28 ± 0.25, which the difference was not statistically significant (*p* = 0.294) (Table S20 and Figure S20).

### 3.21. The OS Rate of the Studied Patients According to Hormone Therapy

In the examination of the hormone therapy of the studied patients, it was found that 852 people had hormone therapy and 194 people did not have chemotherapy. The hormone therapy status of 24 patients was not known. There were 55 deaths among people who had hormone therapy, 16 deaths among people who did not have hormone therapy, and 4 deaths among people whose hormone therapy was not known. The average OS in patients with hormone therapy was 17.06 ± 0.51, and in patients without chemotherapy, it was 18.57 ± 0.77, which was statistically significant (*p* = 0.005) (Table S21 and Figure S21).

### 3.22. The Rate of DFS in the Studied Patients According to Hormone Therapy

In the examination of the hormone therapy of the studied patients, it was found that 854 people had hormone therapy and 195 people did not have chemotherapy. The hormone therapy status of 24 patients was not known. Among the people who had hormone therapy, 92 cases were recorded; among the people who did not have hormone therapy, 29 cases were recorded; and among the people whose hormone therapy was unknown, 2 cases (recurrence, metastasis, and death) were recorded. The OS average was 16.08 ± 0.52 in patients with hormone therapy and 17.08 ± 0.82 in patients without chemotherapy, and this difference was statistically significant (*p* = 0.044) (Table S22 and Figure S22).

### 3.23. The OS Rate of the Studied Patients According to Tumor Size

In examining the tumor size of the patients examined in the study, it was found that the tumor size of 378 people was less than 2 cm, 501 people were between 2 and 5 cm, and 151 people were more than 5 cm, and the tumor size was unknown in 44 patients. Among the patients whose tumor size was less than 2 cm, there were 35 cases; among the patients whose tumor size was between 2 and 5 cm, there were 57 cases; and among the patients whose tumor size was more than 5 cm, there were 28 cases. And among people whose tumor size was unknown, three deaths were recorded. The OS average was 19.88 ± 0.64 in masses below 2 cm, 17.81 ± 0.58 in masses between 2 and 5 cm, and 13.36 ± 1.18 in masses greater than 5 cm, which was statistically significant (*p* ≤ 0.001) (Table S23 and Figure S23).

### 3.24. The DFS Rate in Studied Patients According to Tumor Size

In examining the tumor size of the patients examined in the study, it was found that the tumor size of 378 people was less than 2 cm, 501 people were between 2 and 5 cm, and 151 people were more than 5 cm, and the tumor size was unknown in 44 patients. Among the patients whose tumor size was less than 2 cm, there were 35 cases; among the patients whose tumor size was between 2 and 5 cm, there were 57 cases; and among the patients whose tumor size was more than 5 cm, there were 28 cases. Among the people whose tumor size was unknown, three events (recurrence, metastasis, and death) were recorded. The OS average was 18.25 ± 0.71 in masses below 2 cm, 16.85 ± 0.64 in masses between 2 and 5 cm, and 11.98 ± 0.71 in masses greater than 5 cm, which was statistically significant (*p* = 0.018) (Table S24 and Figure S24).

### 3.25. The OS Rate of the Studied Patients According to the Stage of the Disease

In examining the disease stage of the patients examined in the study, it was found that 21 people were in Stage 0, 286 people were in Stage 1, 488 people were in Stage 2, 224 people were in Stage 3, 10 patients were in Stage 4, and 10 patients were unknown. The patients were distributed according to the stage of the disease as follows: 0 cases in stage zero, 21 cases in the first stage, 51 cases in the second stage, 44 cases in the third stage, and 2 cases in the fourth stage. The average OS of patients according to disease stage can be seen in Table S25 and Figure S25. The difference in OS was significant from the point of view of the chi‐square test (*p* ≤ 0.001).

### 3.26. The Rate of DFS in the Studied Patients According to the Stage of the Disease

The average DFS of patients according to the stage of the disease can be seen in Table S26 and Figure S26. Disease‐related outcomes, including recurrence, metastasis, or death, were recorded in 1 patient with Stage 0 disease, 8 patients with Stage I disease, 31 patients with Stage II disease, 32 patients with Stage III disease, and 2 additional cases (recurrence, metastasis, and death). The difference in mean DFS was significant from the point of view of the chi‐square test (*p* ≤ 0.001).

### 3.27. The OS Rate of the Studied Patients According to Pathology

In examining the pathology of the patients examined in the study, it was found that 756 people had ductal carcinoma in situ (DCIS) and 315 had other cases. Among patients with DCIS, 67 cases were recorded, and among patients with other cases, eight deaths were recorded. The average OS in DCIS was 17.19 ± 0.5 and, in other cases, 18.29 ± 0.34, which the difference was statistically significant (*p* = 0.025) (Table S27 and Figure S27).

Although DCIS is classified as Stage 0, the pathology‐based and stage‐based classifications in our retrospective dataset were recorded independently. Some patients with DCIS pathology had incomplete staging information or were categorized differently due to variations in historical classification systems over the 30‐year study period. Additionally, survival estimates were influenced by different follow‐up durations and censoring patterns. These factors likely contributed to the observed discrepancy between stage‐based and pathology‐based survival estimates.

### 3.28. The Rate of DFS in the Studied Patients According to Pathology

Among the patients with DCIS, 95 cases were recorded, and 28 events (recurrence, metastasis, and death) were recorded among the patients with other cases. The mean DFS in DCIS was 17.19 ± 0.5 and, in other cases, 18.29 ± 0.34, which the difference was not statistically significant (*p* = 0.893) (Table S28 and Figure S28).

Additional subgroup analyses, including survival outcomes according to gender, family history, tumor characteristics, and treatment variables, are presented in Tables S3, S4, S5, S6, S7, S8, S9, S10, S11, S12, S13, S14, S15, S16, S17, S18, S19, S20, S21, S22, S23, S24, S25, S26, S27, S28 and Figures S3, S4, S5, S6, S7, S8, S9, S10, S11, S12, S13, S14, S15, S16, S17, S18, S19, S20, S21, S22, S23, S24, S25, S26, S27, S28.

## 4. Discussion

Breast cancer constitutes 12.5% of all cancers in Iran. It ranks as the sixth primary cause of mortality in the country (11, 12). ALND was the conventional approach used in the surgical treatment of breast cancer patients and the standard technique for axillary lymph node staging some years ago. ALND has been integral to breast cancer surgery since the introduction of the radical mastectomy (13). ALND consistently detects nodal metastases and maintains regional control (14, 15). However, the role of local treatment in breast cancer survival remains contentious. Nonetheless, ALND entails a significant and sometimes intolerable risk of sequelae, including lymphedema, seroma development, and infection (16).

Consequently, SLNB has provided new opportunities for the precise staging of tumor‐draining axillary nodes with reduced consequences compared to ALND. SLNB facilitates precise staging by directing pathologists’ attention towards the nodes most likely to have metastases. Furthermore, SLNB identifies patients who are most likely to benefit from the procedure (those with confirmed regional nodal metastases) and spares those unlikely to benefit from ALND (those with negative SLNs) from the associated morbidity of ALND (17). The Z0011 (18) study conducted by the American College of Surgeons Oncology Group is a Phase 3 multicenter experiment that sparked controversy by questioning the need for ALND when the SLN is positive. A propensity score‐matched investigation of patients with breast cancer with SLN micrometastases in the American Surveillance, Epidemiology, and End Results database (19) revealed no survival difference between those who had axillary dissection and those who received SLNB alone. In this investigation, the average OS and DFS in ALND exceeded those in SLNB; nevertheless, the differences were not statistically significant. The findings of this study aligned with the research conducted by Canavese et al., Veronesi et al., Zavagno et al., Krag et al. (20), Giuliano et al., Tinterri et al., and Gao et al. (18, 21–25). Canavese’s research findings align with the notion that SLNB is an effective method for staging early breast cancer in patients with tumors measuring less than 3 cm at preoperative diagnosis and exhibiting clinically node‐negative illness. The low recurrence rate in the SLNB group, similar to that seen after standard ALND, bolsters the growing view that total axillary dissection may be safely avoided in SLN‐negative individuals, therefore alleviating them from the recognized acute complications of axillary surgery. Veronesi et al.’s research indicates that SLNB might prevent the need for complete ALND in patients with negative SLNs, hence decreasing postoperative morbidity and hospital stay expenses (23).

Tinterri et al. demonstrated that in patients with T1–2 breast cancer and one to two macrometastatic SLNs who underwent mastectomy, the OS and recurrence‐free survival rates for those treated with SLNB alone were not inferior to those who received ALND (22). Gao et al.’s research indicated that ALND may be unnecessary for patients with one to two positive SLNs who had a complete mastectomy (24). Galimberti et al. (26) presented a 5‐year follow‐up of the randomized IBSCG 23‐01 study, revealing a 5‐year DFS rate of 87.8% (95% confidence interval: 84.4–91.2) in the cohort without axillary dissection, with no statistically significant difference in survival between the groups. The latter was subsequently corroborated by a decade of data from the same population (27).

The studies calculated and compared the odds ratios for OS and DFS between two patient groups undergoing ALND and SLNB, revealing no significant statistical difference between the odds ratios of the ALND and SLNB groups in any of the studies. The primary objective of this study was the comparison between ALND and SLNB. Other subgroup analyses should be interpreted cautiously, as they were exploratory and not adjusted for multiple comparisons.

Our findings are consistent with recent systematic reviews and meta‐analyses demonstrating no significant survival advantage of ALND compared with SLNB in appropriately selected breast cancer patients. A recent meta‐analysis confirmed that omission of ALND in patients undergoing SLNB does not compromise OS or DFS, while significantly reducing surgical morbidity (28). These findings further support the ongoing de‐escalation of axillary surgery in modern breast cancer management.

This study found that OS and DFS rates were markedly higher in individuals who underwent BCS compared to those who received MRM or MRM combined with BCS, which contradicts the findings of Daryabor et al. and Singletary et al.’s studies (29, 30).

Daryabor et al.’s research indicates that BCS did not elevate the risk of locoregional recurrence or metastasis or affect the survival rate compared to MRM. Therefore, taking into account the esthetic outcomes and postoperative problems, BCS is a superior method (29).

Singletary et al.’s research demonstrated that local recurrence rates were minimal and DFS rates were significantly elevated in both the conservation treatment and mastectomy groups across all diagnostic years and all pathologic tumor size/lymph node status classifications (30).

These trials indicated no advantage of BCS over MRM regarding survival; nevertheless, a significant aspect was the surgeons’ preference for selecting the surgical approach based on the illness stage. In these investigations, surgeons performed large operations on patients in the early stages of the illness using the BCS approach, whereas patients in the advanced stages underwent the MRM procedure. The patients undergoing the MRM technique in the present research may be at more advanced stages of the illness, resulting in a poorer survival rate.

The single ER+ and PR+ subtypes comprised about 10% of all molecular breast cancer subtypes (31).

Certain investigations indicated no prognostic differences between ER + PR− and ER − PR+ individuals (32, 33). Other studies indicated ER − PR+ patients had a worse prognosis than ER + PR− cases (31, 34). Our investigation found no correlation between DFS and OS concerning ER and PR hormone receptors, as well as P53 and HER2 genes, aligning with the results of Daryabor et al. (29) and Swenson et al. (35). Swenson et al.’s investigation indicated that negative progesterone‐receptor status was not a significant factor for local recurrence (35).

Our research findings indicated that, as anticipated, OS was much greater in individuals who had chemotherapy than in those who did not get chemotherapy or who received neoadjuvant chemotherapy.

Tamoxifen is a commonly used hormone‐based medication for breast cancer in both adjuvant and metastatic contexts, extending overall and recurrence‐free survival (36). Tamoxifen functions as a selective estrogen receptor modulator (SERM), inhibiting the transcriptional activity of the estrogen receptor by competitive binding (37). Due to ongoing enhancements in DFS and OS shown in subsequent meta‐analyses (38), tamoxifen has served as a principal therapy modality for breast cancer, especially among premenopausal women, for more than 40 years. Among women aged 60 years and older who had adjuvant tamoxifen without axillary therapy, the incidence of axillary recurrence was just 3%, and the OS rate was 73% at a median follow‐up of 6.6 years (39). The findings of our investigation unexpectedly revealed no correlation between OS and DFS regarding hormone treatment.

## 5. Limitations

A major limitation of this study is the lack of adjustment for potential confounding variables such as disease stage, molecular subtype, and treatment era. Because of limitations related to retrospective data structure and available statistical resources, multivariable Cox regression analysis and propensity score matching could not be performed. Therefore, the observed survival comparisons between ALND and SLNB groups may be influenced by baseline differences between groups. In particular, patients undergoing ALND may have had more advanced disease or been treated during earlier therapeutic eras, which may affect survival outcomes independently of surgical approach.

Additionally, the long study period (1991–2021) encompasses substantial advances in imaging, pathological assessment, systemic therapy, and surgical management of breast cancer. These temporal changes represent an additional source of confounding that could not be fully controlled in the present analysis. An important limitation of this study is the extended study period from 1991 to 2021, which spans major advances in breast cancer diagnosis and treatment. Over these three decades, significant improvements have occurred in imaging techniques, pathological evaluation, surgical techniques, systemic chemotherapy, endocrine therapy, and targeted therapies such as anti‐HER2 agents. These temporal changes may have influenced survival outcomes independently of the type of axillary surgery and represent a potential source of confounding. Furthermore, SLNB became more widely adopted in later years, when systemic therapies were more effective, which could bias comparisons between groups. To mitigate these effects, adjusted and propensity score analyses were performed; however, residual confounding cannot be fully excluded. The retrospective design, lack of randomization, and long study period introduce potential selection bias and confounding. Patients undergoing ALND may have had more advanced disease, while those undergoing SLNB may have been treated in more recent periods with shorter follow‐up durations. Additionally, treatment approaches, systemic therapy, and diagnostic techniques evolved significantly over the study period. These factors may have influenced survival outcomes independently of surgical technique.

Selection bias is an important consideration in this retrospective study. Patients undergoing ALND may have had more advanced disease characteristics, which could influence survival independently of surgical approach.

## 6. Conclusions

In this large retrospective cohort, no statistically significant differences in OS or DFS were observed between patients undergoing ALND and SLNB after adjustment for confounding variables and propensity score matching. These findings support the oncologic safety of SLNB as an alternative to ALND in appropriately selected patients. However, the retrospective design and long study period remain important limitations.

## Author Contributions

Mahta Mellat Ardakani and Bahar Amiri contributed equally to this work and should be considered co‐first authors.

## Funding

No funding was received for this manuscript.

## Conflicts of Interest

The authors declare no conflicts of interest.

## Supporting Information

Additional supporting information can be found online in the Supporting Information section.

## Supporting information


**Supporting Information 1** Table S3 shows a comparison of the overall survival rate according to gender.

**Supporting Information 2** Figure S3 shows a comparison of the overall survival rate according to.

**Supporting Information 3** Table S4 shows a comparison of the disease‐free survival rate according to gender.

**Supporting Information 4** Figure S4 shows a comparison of the disease‐free survival rate according to gender.

**Supporting Information 5** Table S5 shows a comparison of the overall survival rate according to family history.

**Supporting Information 6** Figure S5 shows a comparison of the overall survival rate according to family history.

**Supporting Information 7** Table S6 shows a comparison of the disease‐free survival rate according to family history.

**Supporting Information 8** Figure S6 shows a comparison of the disease‐free survival rate according to family history.

**Supporting Information 9** Table S7 shows a comparison of the overall survival rate according to the type of surgery.

**Supporting Information 10** Figure S7 shows a comparison of the overall survival rate according to the type of surgery.

**Supporting Information 11** Table S8 shows a comparison of the disease‐free survival rate according to the type of surgery.

**Supporting Information 12** Figure S8 shows a comparison of the disease‐free survival rate according to the type of surgery.

**Supporting Information 13** Table S9 shows a comparison of the overall survival rate according to lymphatic vascular invasion.

**Supporting Information 14** Figure S9 shows a comparison of the overall survival rate according to lymphatic vascular invasion.

**Supporting Information 15** Table S10 shows a comparison of the disease‐free survival rate according to lymphatic vascular invasion.

**Supporting Information 16** Figure S10 shows a comparison of the disease‐free survival rate according to lymphatic vascular invasion.

**Supporting Information 17** Table S11 shows a comparison of the overall survival rate according to the presence of the ER hormone receptor.

**Supporting Information 18** Figure S11 shows a comparison of the overall survival rate according to the presence of the ER hormone receptor.

**Supporting Information 19** Table S12 shows a comparison of the disease‐free survival rate according to the presence of the ER hormone receptor.

**Supporting Information 20** Figure S12 shows a comparison of the disease‐free survival rate according to the presence of the ER hormone receptor.

**Supporting Information 21** Table S13 shows a comparison of the overall survival rate according to the presence of the PR hormone receptor.

**Supporting Information 22** Figure S13 shows a comparison of the overall survival rate according to the presence of the PR hormone receptor.

**Supporting Information 23** Table S14 shows a comparison of the disease‐free survival rate according to the presence of the PR hormone receptor.

**Supporting Information 24** Figure S14 shows a comparison of the disease‐free survival rate according to the presence of the PR hormone receptor.

**Supporting Information 25** Table S15 shows a comparison of the overall survival rate according to the presence of the P53 gene.

**Supporting Information 26** Figure S15 shows a comparison of the overall survival rate according to the presence of the P53 gene.

**Supporting Information 27** Table S16 shows a comparison of the disease‐free survival rate according to the presence of the P53 gene.

**Supporting Information 28** Figure S16 shows a comparison of the disease‐free survival rate according to the presence of the P53 gene.

**Supporting Information 29** Table S17 shows a comparison of the overall survival rate according to the presence of the HER‐2 gene.

**Supporting Information 30** Figure S17 shows a comparison of the overall survival rate according to the presence of the HER‐2 gene.

**Supporting Information 31** Table S18 shows a comparison of the disease‐free survival rate according to the presence of the HER‐2 gene.

**Supporting Information 32** Figure S18 shows a comparison of the disease‐free survival rate according to the presence of the HER‐2 gene.

**Supporting Information 33** Table S19 shows a comparison of the overall survival rate according to chemotherapy.

**Supporting Information 34** Figure S19 shows a comparison of the overall survival rate according to chemotherapy.

**Supporting Information 35** Table S20 shows a comparison of the disease‐free survival rate according to chemotherapy.

**Supporting Information 36** Figure S20 shows a comparison of the disease‐free survival rate according to chemotherapy.

**Supporting Information 37** Table S21 shows a comparison of the overall survival rate according to hormone therapy.

**Supporting Information 38** Figure S21 shows a comparison of the overall survival rate according to hormone therapy.

**Supporting Information 39** Table S22 shows a comparison of the disease‐free survival rate according to hormone therapy.

**Supporting Information 40** Figure S22 shows a comparison of the disease‐free survival rate according to hormone therapy.

**Supporting Information 41** Table S23 shows a comparison of the overall survival rate according to tumor size.

**Supporting Information 42** Figure S23 shows a comparison of the overall survival rate according to tumor size.

**Supporting Information 43** Table S24 shows a comparison of the disease‐free survival rate according to tumor size.

**Supporting Information 44** Figure S24 shows a comparison of the disease‐free survival rate according to tumor size.

**Supporting Information 45** Table S25 shows a comparison of the overall survival rate according to the stage of the disease.

**Supporting Information 46** Figure S25 shows a comparison of the overall survival rate according to the stage of the disease.

**Supporting Information 47** Table S26 shows a comparison of the disease‐free survival rate according to the stage of the disease.

**Supporting Information 48** Figure S26 shows a comparison of the disease‐free survival rate according to the stage of the disease.

**Supporting Information 49** Table S27 shows a comparison of the overall survival rate according to pathology.

**Supporting Information 50** Figure S27 shows a comparison of the overall survival rate according to pathology.

**Supporting Information 51** Table S28 shows a comparison of the disease‐free survival rate according to tumor size.

**Supporting Information 52** Figure S28 shows a comparison of the disease‐free survival rate according to tumor size.

## Data Availability

The data that support the findings of this study are available from the corresponding author upon reasonable request.
