# Supplementary material for: Comparing Disease‐Free Survival (DFS) and Overall Survival (OS) Rates in Breast Cancer Patients: Axillary Lymph Node Dissection (ALND) Versus Sentinel Lymph Node Biopsy (SLNB)
Source: Int J Breast Cancer. 2026 Jun 26;2026:5039446. doi: 10.1155/ijbc/5039446 (PMC13305675; doi:10.1155/ijbc/5039446)
Supplement: Supplementary file 1 — Supporting Information 1 Table S3 shows a comparison of the overall survival rate according to gender. [file IJBC-2026-5039446-s036.docx]

| **Supplementary Table S3: Comparison of overall survival rate according to gender (P = 0.07)** | | | | |
| --- | --- | --- | --- | --- |
| Gender | Average | Standard deviation | 95 percent confidence interval | |
|  |  |  | Lower bound | Upper bound |
| ALND | 18.207 | 0.506 | 17.214 | 19.200 |
| SLNB | 10.143 | 2.839 | 4.578 | 15.707 |
