# Supplementary material for: Comparing Disease‐Free Survival (DFS) and Overall Survival (OS) Rates in Breast Cancer Patients: Axillary Lymph Node Dissection (ALND) Versus Sentinel Lymph Node Biopsy (SLNB)
Source: Int J Breast Cancer. 2026 Jun 26;2026:5039446. doi: 10.1155/ijbc/5039446 (PMC13305675; doi:10.1155/ijbc/5039446)
Supplement: Supplementary file 2 — Supporting Information 2 Figure S3 shows a comparison of the overall survival rate according to. [file IJBC-2026-5039446-s037.docx]

Survival Functions

TIME.DEATH.YEAR

Gender

Female Male

Cencored-Female Cencored-Male


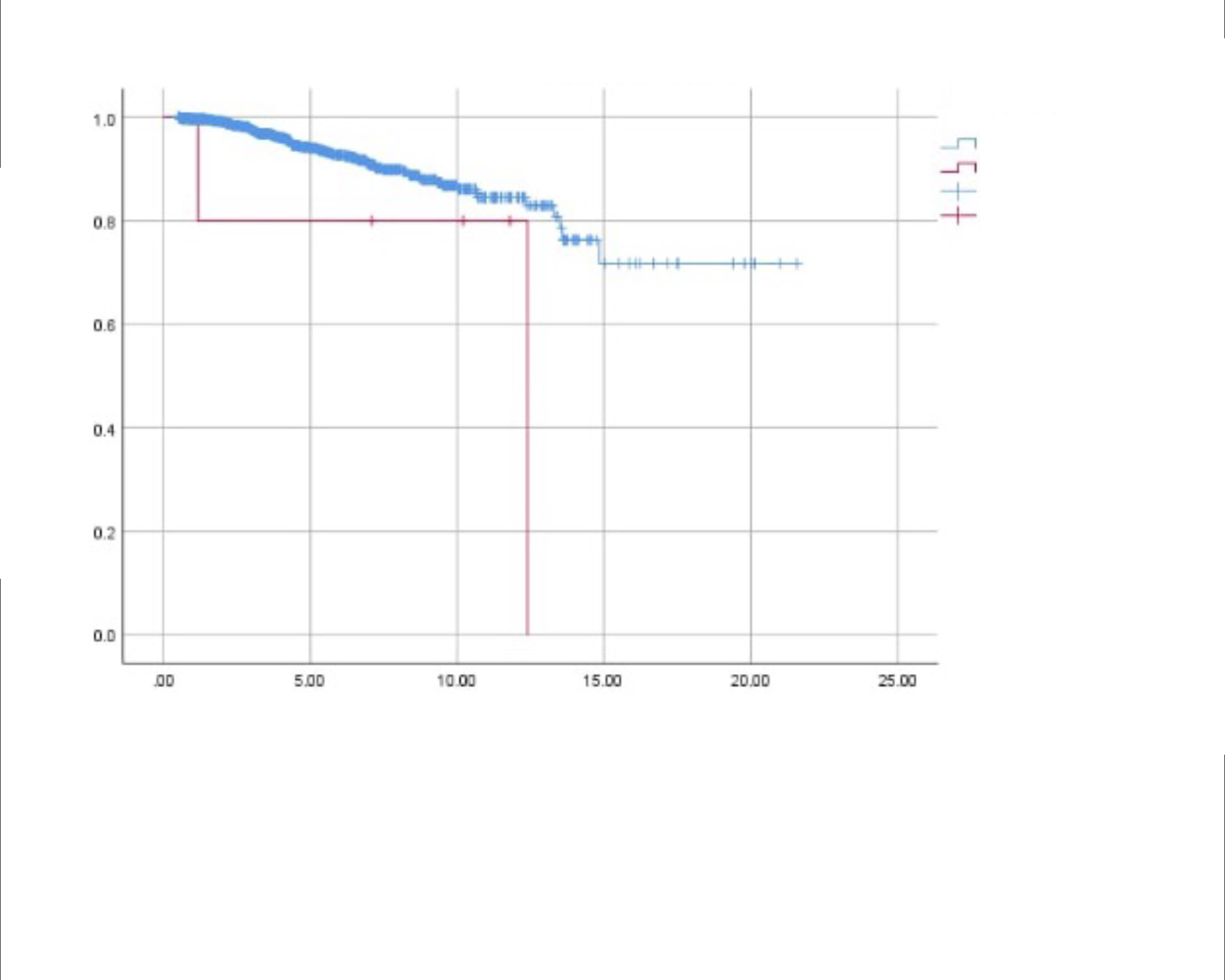


C u m S u r v i v a l

Supplementary Figure S3: Comparison of overall survival rate according to gender (P = 0.07)
