# Supplementary material for: Comparing Disease‐Free Survival (DFS) and Overall Survival (OS) Rates in Breast Cancer Patients: Axillary Lymph Node Dissection (ALND) Versus Sentinel Lymph Node Biopsy (SLNB)
Source: Int J Breast Cancer. 2026 Jun 26;2026:5039446. doi: 10.1155/ijbc/5039446 (PMC13305675; doi:10.1155/ijbc/5039446)
Supplement: Supplementary file 3 — Supporting Information 3 Table S4 shows a comparison of the disease‐free survival rate according to gender. [file IJBC-2026-5039446-s038.docx]

| **Supplementary Table S4: Comparison of disease-free survival rate according to gender (P = 0.855)** | | | | |
| --- | --- | --- | --- | --- |
| Gender | Average | Standard deviation | 95 percent confidence interval | |
|  |  |  | Lower bound | Upper bound |
| ALND | 17.190 | 0.513 | 16.185 | 18.195 |
| SLNB | 11.295 | 0.349 | 10.612 | 11.979 |
