# Supplementary material for: Comparing Disease‐Free Survival (DFS) and Overall Survival (OS) Rates in Breast Cancer Patients: Axillary Lymph Node Dissection (ALND) Versus Sentinel Lymph Node Biopsy (SLNB)
Source: Int J Breast Cancer. 2026 Jun 26;2026:5039446. doi: 10.1155/ijbc/5039446 (PMC13305675; doi:10.1155/ijbc/5039446)
Supplement: Supplementary file 4 — Supporting Information 4 Figure S4 shows a comparison of the disease‐free survival rate according to gender. [file IJBC-2026-5039446-s039.docx]

Survival Functions

Gender


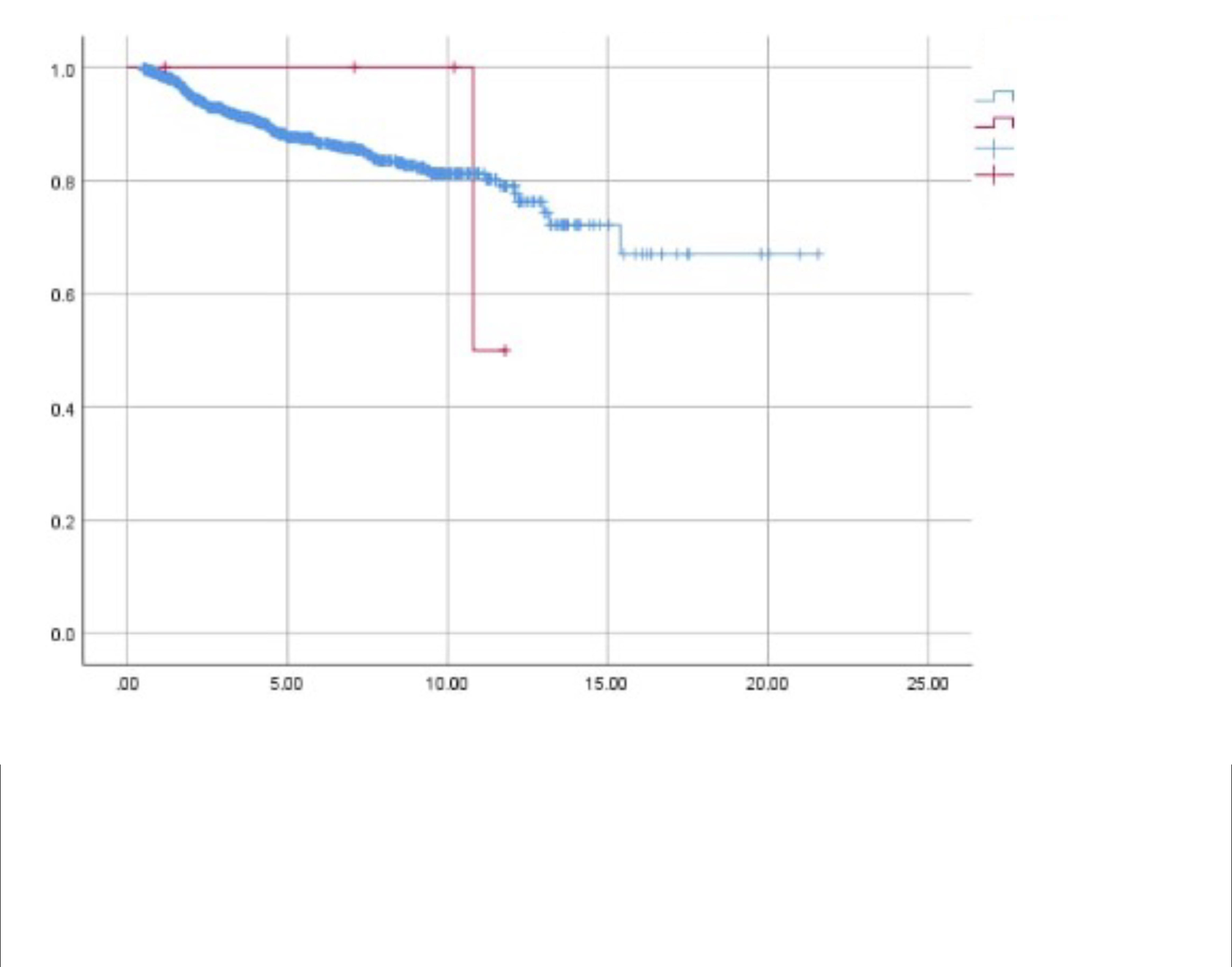


C u m S u r v i v a l

Female Male

Cencored-Female Cencored-Male

TIME.REC.YEAR

Supplementary Figure S4: Comparison of disease-free survival rate according to gender (P= 0.855)
