# Supplementary material for: Comparing Disease‐Free Survival (DFS) and Overall Survival (OS) Rates in Breast Cancer Patients: Axillary Lymph Node Dissection (ALND) Versus Sentinel Lymph Node Biopsy (SLNB)
Source: Int J Breast Cancer. 2026 Jun 26;2026:5039446. doi: 10.1155/ijbc/5039446 (PMC13305675; doi:10.1155/ijbc/5039446)
Supplement: Supplementary file 5 — Supporting Information 5 Table S5 shows a comparison of the overall survival rate according to family history. [file IJBC-2026-5039446-s040.docx]

| **Supplementary Table S5: Comparison of overall survival rate according to family history (P = 0.63)** | | | | |
| --- | --- | --- | --- | --- |
| Family history | Average | Standard deviation | 95 percent confidence interval | |
|  |  |  | Lower bound | Upper bound |
| Negative | 16.635 | 0.626 | 15.409 | 17.861 |
| First degree relative | 14.410 | 0.370 | 13.686 | 15.135 |
| Second degree relative | 18.393 | 1.065 | 16.306 | 20.480 |
