# Supplementary material for: Comparing Disease‐Free Survival (DFS) and Overall Survival (OS) Rates in Breast Cancer Patients: Axillary Lymph Node Dissection (ALND) Versus Sentinel Lymph Node Biopsy (SLNB)
Source: Int J Breast Cancer. 2026 Jun 26;2026:5039446. doi: 10.1155/ijbc/5039446 (PMC13305675; doi:10.1155/ijbc/5039446)
Supplement: Supplementary file 6 — Supporting Information 6 Figure S5 shows a comparison of the overall survival rate according to family history. [file IJBC-2026-5039446-s051.docx]

# Survival Functions

Family history


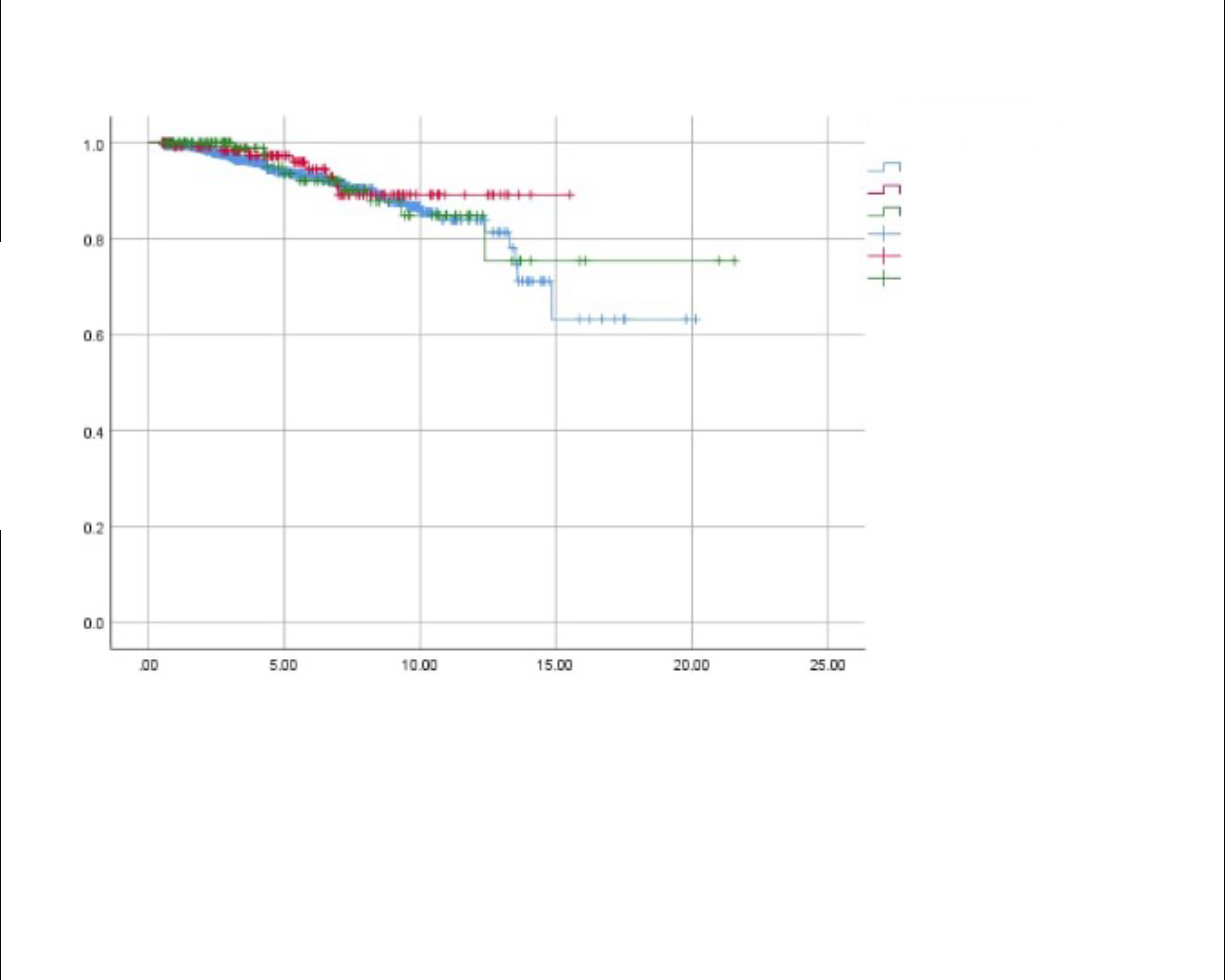


C u m S u r v i v a l

Negative

First degree relative Second degree relative censored- Negative

censored- First degree relative censored- Second degree relative

TIME.DEATH.YEAR

Supplementary Figure S5: Comparison of overall survival rate according to family history (P= 0.63)
