# Supplementary material for: Comparing Disease‐Free Survival (DFS) and Overall Survival (OS) Rates in Breast Cancer Patients: Axillary Lymph Node Dissection (ALND) Versus Sentinel Lymph Node Biopsy (SLNB)
Source: Int J Breast Cancer. 2026 Jun 26;2026:5039446. doi: 10.1155/ijbc/5039446 (PMC13305675; doi:10.1155/ijbc/5039446)
Supplement: Supplementary file 7 — Supporting Information 7 Table S6 shows a comparison of the disease‐free survival rate according to family history. [file IJBC-2026-5039446-s052.docx]

| **Supplementary Table S6: Comparison of disease-free survival rate according to family history (P = 0.26)** | | | | |
| --- | --- | --- | --- | --- |
| family history | Average | Standard deviation | 95 percent confidence interval | |
|  |  |  | Lower bound | Upper bound |
| Negative | 15.293 | 0.585 | 14.146 | 15.293 |
| First degree relative | 13.766 | 0.523 | 12.742 | 13.766 |
| Second degree relative | 18.127 | 0.784 | 16.590 | 18.127 |
