# Supplementary material for: Comparing Disease‐Free Survival (DFS) and Overall Survival (OS) Rates in Breast Cancer Patients: Axillary Lymph Node Dissection (ALND) Versus Sentinel Lymph Node Biopsy (SLNB)
Source: Int J Breast Cancer. 2026 Jun 26;2026:5039446. doi: 10.1155/ijbc/5039446 (PMC13305675; doi:10.1155/ijbc/5039446)
Supplement: Supplementary file 8 — Supporting Information 8 Figure S6 shows a comparison of the disease‐free survival rate according to family history. [file IJBC-2026-5039446-s050.docx]

# Survival Functions

Family history


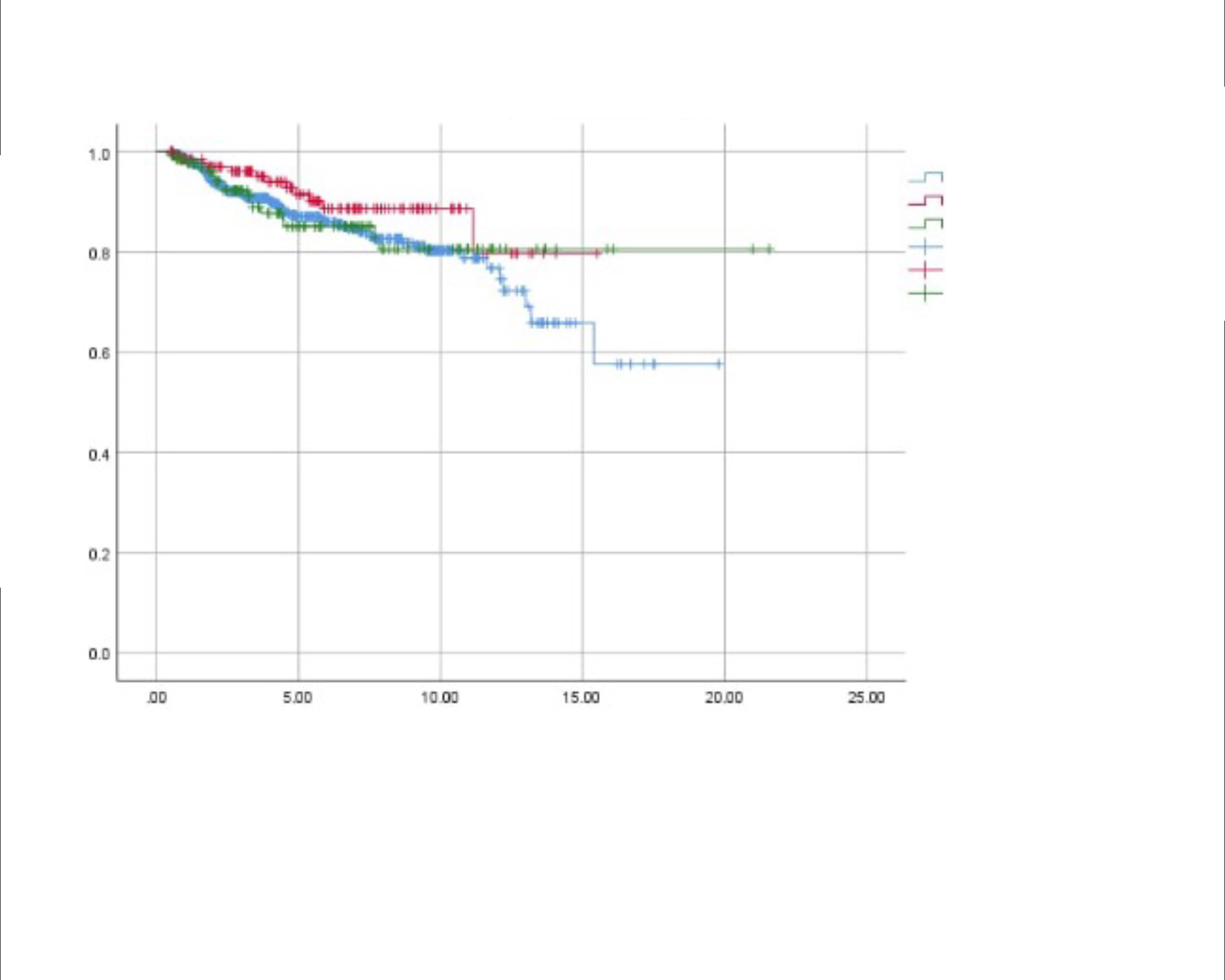


C u m S u r v i v a l

Negative

First degree relative Second degree relative censored- Negative

censored- First degree relative censored- Second degree relative

# TIME.REC.YEAR

Supplementary Figure S6: Comparison of disease-free survival rate according to family history (P = 0.26)
