# Supplementary material for: Comparing Disease‐Free Survival (DFS) and Overall Survival (OS) Rates in Breast Cancer Patients: Axillary Lymph Node Dissection (ALND) Versus Sentinel Lymph Node Biopsy (SLNB)
Source: Int J Breast Cancer. 2026 Jun 26;2026:5039446. doi: 10.1155/ijbc/5039446 (PMC13305675; doi:10.1155/ijbc/5039446)
Supplement: Supplementary file 9 — Supporting Information 9 Table S7 shows a comparison of the overall survival rate according to the type of surgery. [file IJBC-2026-5039446-s049.docx]

| **Supplementary Table S7: Comparison of overall survival rate according to the type of surgery (P≤0.001)** | | | | |
| --- | --- | --- | --- | --- |
| Type of surgery | Average | Standard deviation | 95 percent confidence interval | |
|  |  |  | Lower bound | Upper bound |
| Unknown | 10.115 | 0.475 | 9.184 | 11.046 |
| BCS | 19.317 | 0.451 | 18.433 | 20.200 |
| MRM | 15.737 | 0.880 | 14.011 | 17.462 |
| BCS/MRM | 11.545 | 1.798 | 8.020 | 15.069 |
