# Supplementary material for: Comparing Disease‐Free Survival (DFS) and Overall Survival (OS) Rates in Breast Cancer Patients: Axillary Lymph Node Dissection (ALND) Versus Sentinel Lymph Node Biopsy (SLNB)
Source: Int J Breast Cancer. 2026 Jun 26;2026:5039446. doi: 10.1155/ijbc/5039446 (PMC13305675; doi:10.1155/ijbc/5039446)
Supplement: Supplementary file 10 — Supporting Information 10 Figure S7 shows a comparison of the overall survival rate according to the type of surgery. [file IJBC-2026-5039446-s048.docx]

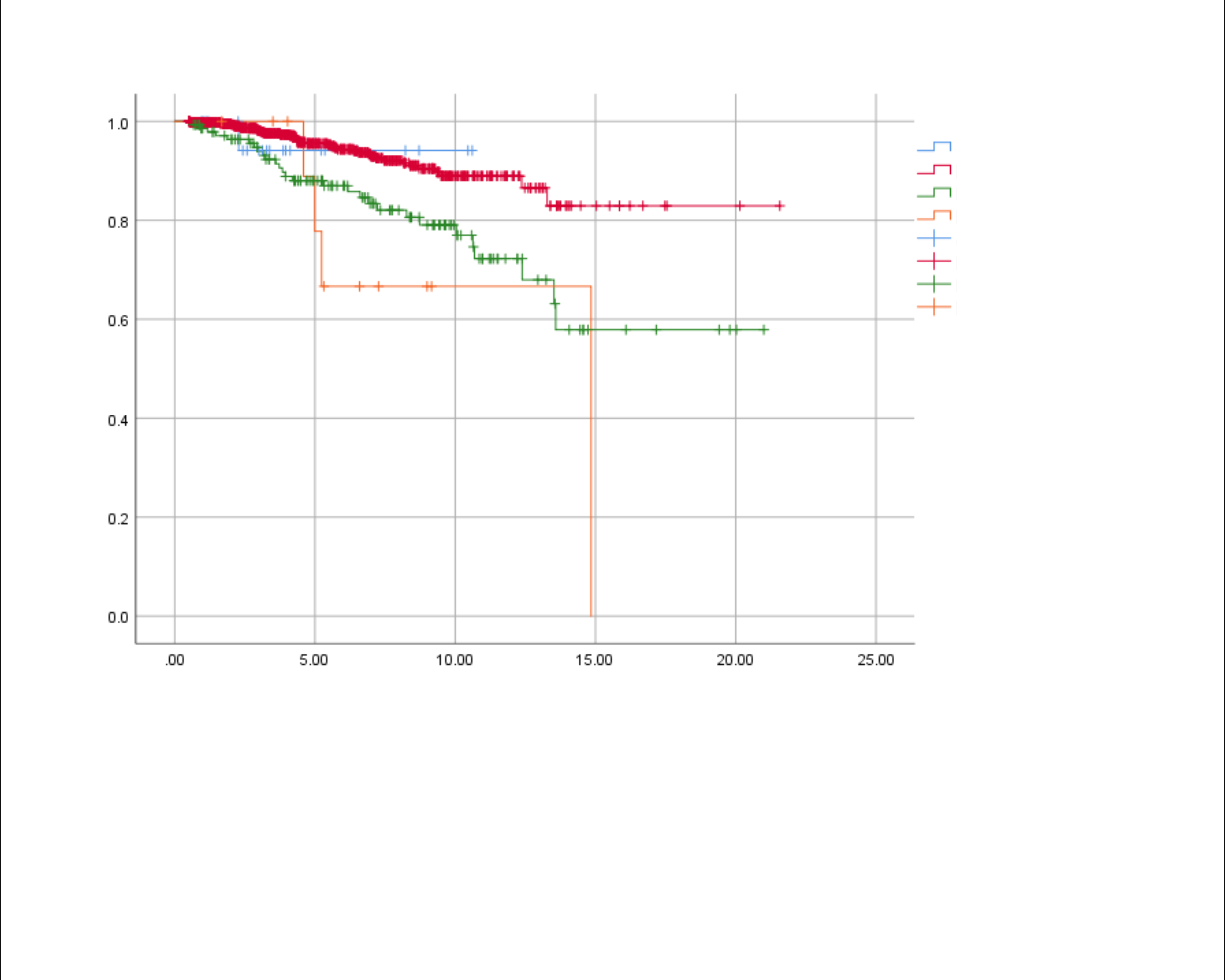
Survival Functions Type of surgery

C u m S u r v i v a l

Unknown BCS

MRM BCS/MRM

censored- Unknown BCS-censored

MRM-censored BCS/MRM-censored

TIME.DEATH.YEAR

Supplementary Figure S7: Comparison of overall survival rate according to the type of surgery (P≤0.001)
