# Supplementary material for: Comparing Disease‐Free Survival (DFS) and Overall Survival (OS) Rates in Breast Cancer Patients: Axillary Lymph Node Dissection (ALND) Versus Sentinel Lymph Node Biopsy (SLNB)
Source: Int J Breast Cancer. 2026 Jun 26;2026:5039446. doi: 10.1155/ijbc/5039446 (PMC13305675; doi:10.1155/ijbc/5039446)
Supplement: Supplementary file 11 — Supporting Information 11 Table S8 shows a comparison of the disease‐free survival rate according to the type of surgery. [file IJBC-2026-5039446-s047.docx]

| **Supplementary Table S8: Comparison of disease-free survival rate according to the type of surgery (P≤0.001)** | | | | |
| --- | --- | --- | --- | --- |
| type of surgery | Average | Standard deviation | 95 percent confidence interval | |
|  |  |  | Lower bound | Upper bound |
| Unknown | 7.248 | 1.089 | 5.115 | 9.382 |
| BCS | 17.622 | .736 | 16.180 | 19.064 |
| MRM | 15.577 | .842 | 13.926 | 17.228 |
| BCS/MRM | 2.911 | .607 | 1.721 | 4.102 |
