# Supplementary material for: Comparing Disease‐Free Survival (DFS) and Overall Survival (OS) Rates in Breast Cancer Patients: Axillary Lymph Node Dissection (ALND) Versus Sentinel Lymph Node Biopsy (SLNB)
Source: Int J Breast Cancer. 2026 Jun 26;2026:5039446. doi: 10.1155/ijbc/5039446 (PMC13305675; doi:10.1155/ijbc/5039446)
Supplement: Supplementary file 12 — Supporting Information 12 Figure S8 shows a comparison of the disease‐free survival rate according to the type of surgery. [file IJBC-2026-5039446-s046.docx]

Survival Functions

Type of surgery

Unknown BCS

MRM BCS/MRM

censored- Unknown BCS-censored

MRM-censored BCS/MRM-censored


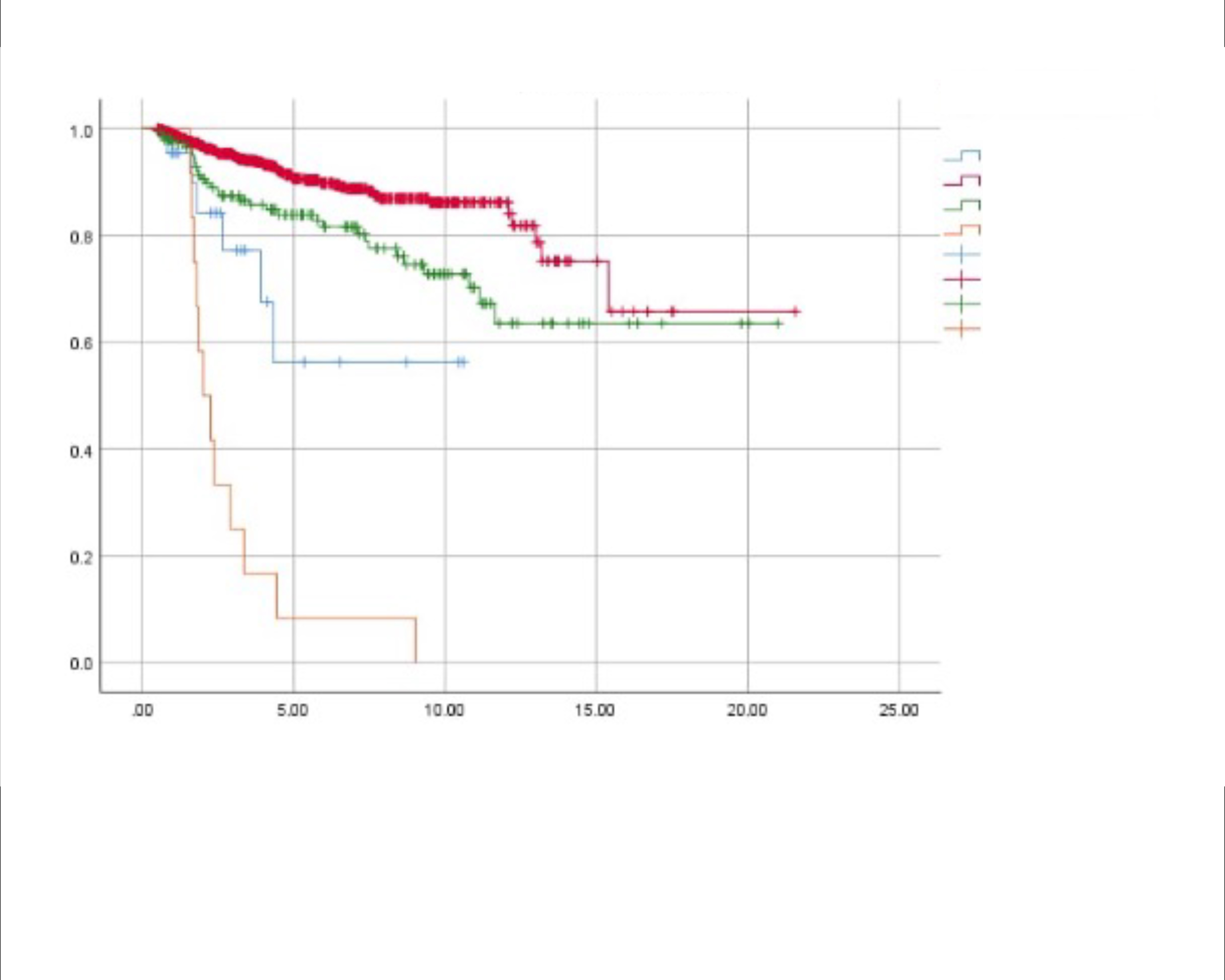


C u m S u r v i v a l

TIME.REC.YEAR

Supplementary Figure S8: Comparison of disease-free survival rate according to the type of surgery (P≤0.001)
