# Supplementary material for: Comparing Disease‐Free Survival (DFS) and Overall Survival (OS) Rates in Breast Cancer Patients: Axillary Lymph Node Dissection (ALND) Versus Sentinel Lymph Node Biopsy (SLNB)
Source: Int J Breast Cancer. 2026 Jun 26;2026:5039446. doi: 10.1155/ijbc/5039446 (PMC13305675; doi:10.1155/ijbc/5039446)
Supplement: Supplementary file 13 — Supporting Information 13 Table S9 shows a comparison of the overall survival rate according to lymphatic vascular invasion. [file IJBC-2026-5039446-s045.docx]

| **Supplementary Table S9: Comparison of overall survival rate according to lymphatic vascular invasion (P≤0.001)** | | | | |
| --- | --- | --- | --- | --- |
| lymphatic vascular invasion | Average | Standard deviation | 95 percent confidence interval | |
|  |  |  | Lower bound | Upper bound |
| Present | 13.217 | 0.711 | 11.824 | 14.611 |
| Unknown | 16.190 | 1.180 | 13.877 | 18.502 |
| Absent | 19.654 | 0.448 | 18.776 | 20.532 |
