# Supplementary material for: Comparing Disease‐Free Survival (DFS) and Overall Survival (OS) Rates in Breast Cancer Patients: Axillary Lymph Node Dissection (ALND) Versus Sentinel Lymph Node Biopsy (SLNB)
Source: Int J Breast Cancer. 2026 Jun 26;2026:5039446. doi: 10.1155/ijbc/5039446 (PMC13305675; doi:10.1155/ijbc/5039446)
Supplement: Supplementary file 15 — Supporting Information 15 Table S10 shows a comparison of the disease‐free survival rate according to lymphatic vascular invasion. [file IJBC-2026-5039446-s043.docx]

| **Supplementary Table S10: Comparison of disease-free survival rate according to lymphatic vascular invasion (P≤0.001)** | | | | |
| --- | --- | --- | --- | --- |
| Lymphatic vascular invasion | Average | Standard deviation | 95 percent confidence interval | |
|  |  |  | Lower bound | Upper bound |
| Present | 12.726 | 0.645 | 11.461 | 13.990 |
| Unknown | 16.611 | 0.831 | 14.982 | 18.240 |
| Absent | 18.025 | 0.715 | 16.624 | 19.426 |
