# Supplementary material for: Comparing Disease‐Free Survival (DFS) and Overall Survival (OS) Rates in Breast Cancer Patients: Axillary Lymph Node Dissection (ALND) Versus Sentinel Lymph Node Biopsy (SLNB)
Source: Int J Breast Cancer. 2026 Jun 26;2026:5039446. doi: 10.1155/ijbc/5039446 (PMC13305675; doi:10.1155/ijbc/5039446)
Supplement: Supplementary file 16 — Supporting Information 16 Figure S10 shows a comparison of the disease‐free survival rate according to lymphatic vascular invasion. [file IJBC-2026-5039446-s042.docx]

Survival Functions

Lymphatic vascular invasion


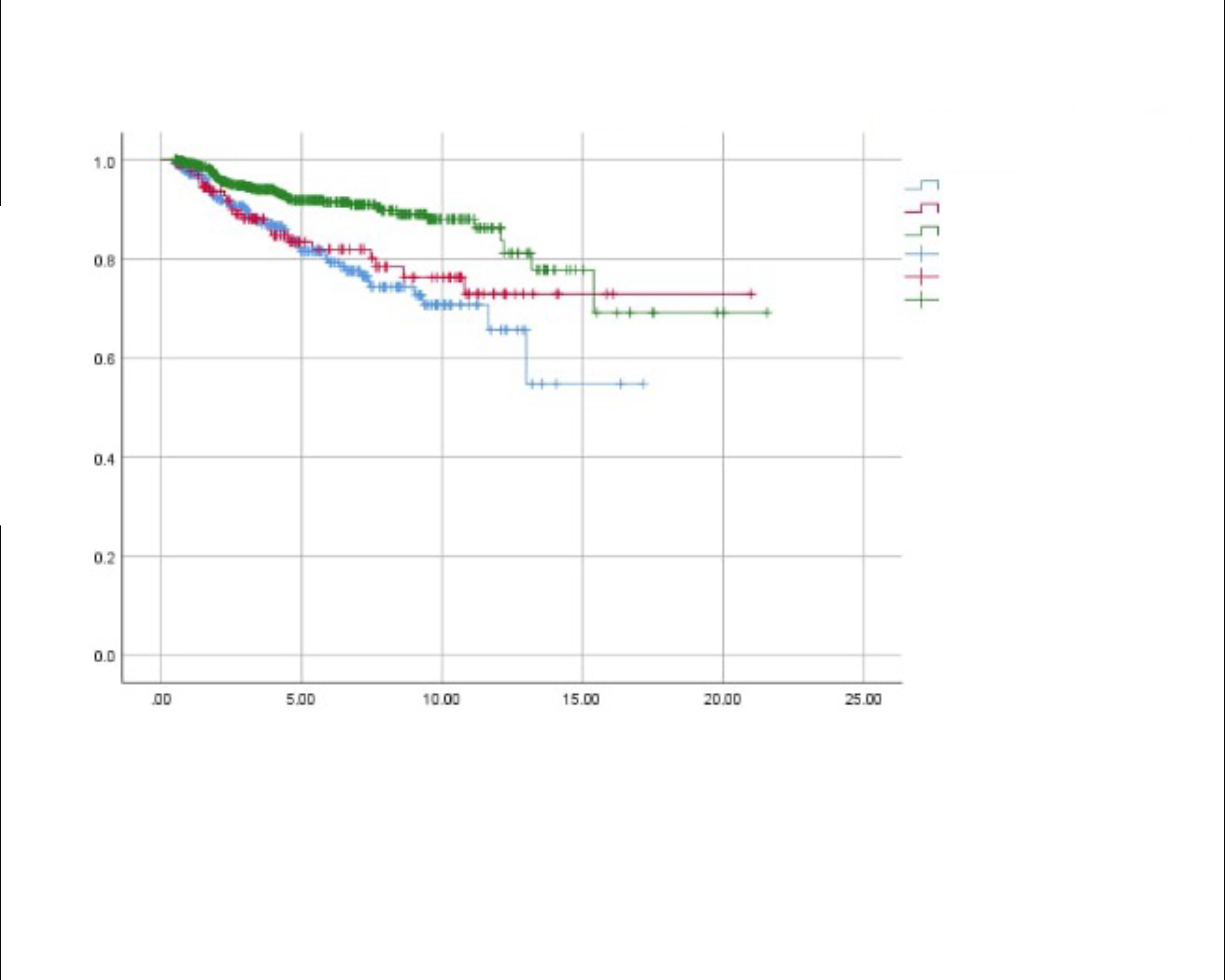


C u m S u r v i v a l

Present Unknown Absent

censored- Present censored- Unknown censored- Absent

TIME.REC.YEAR

Supplementary Figure S10: Comparison of disease-free survival rate according to lymphatic vascular invasion (P≤0.001)
