# Supplementary material for: Comparing Disease‐Free Survival (DFS) and Overall Survival (OS) Rates in Breast Cancer Patients: Axillary Lymph Node Dissection (ALND) Versus Sentinel Lymph Node Biopsy (SLNB)
Source: Int J Breast Cancer. 2026 Jun 26;2026:5039446. doi: 10.1155/ijbc/5039446 (PMC13305675; doi:10.1155/ijbc/5039446)
Supplement: Supplementary file 17 — Supporting Information 17 Table S11 shows a comparison of the overall survival rate according to the presence of the ER hormone receptor. [file IJBC-2026-5039446-s041.docx]

| **Supplementary Table S11: Comparison of overall survival rate according to the presence of the ER hormone receptor (P = 0.07)** | | | | |
| --- | --- | --- | --- | --- |
| ER hormone receptor | Average | Standard deviation | 95 percent confidence interval | |
|  |  |  | Lower bound | Upper bound |
| Present | 16.928 | 0.611 | 15.730 | 18.127 |
| Unknown | 10.406 | 0.737 | 8.961 | 11.851 |
| Absent | 18.439 | 0.585 | 17.294 | 19.585 |
