# Supplementary material for: Comparing Disease‐Free Survival (DFS) and Overall Survival (OS) Rates in Breast Cancer Patients: Axillary Lymph Node Dissection (ALND) Versus Sentinel Lymph Node Biopsy (SLNB)
Source: Int J Breast Cancer. 2026 Jun 26;2026:5039446. doi: 10.1155/ijbc/5039446 (PMC13305675; doi:10.1155/ijbc/5039446)
Supplement: Supplementary file 19 — Supporting Information 19 Table S12 shows a comparison of the disease‐free survival rate according to the presence of the ER hormone receptor. [file IJBC-2026-5039446-s002.docx]

| **Supplementary Table S12: Comparison of disease-free survival rate according to the presence of the ER hormone receptor (P = 0.009)** | | | | |
| --- | --- | --- | --- | --- |
| ER hormone receptor | Average | Standard deviation | 95 percent confidence interval | |
|  |  |  | Lower bound | Upper bound |
| Present | 16.142 | 0.657 | 14.856 | 17.429 |
| Unknown | 11.773 | 0.420 | 10.950 | 12.596 |
| Absent | 16.379 | 0.850 | 14.712 | 18.046 |
