# Supplementary material for: Comparing Disease‐Free Survival (DFS) and Overall Survival (OS) Rates in Breast Cancer Patients: Axillary Lymph Node Dissection (ALND) Versus Sentinel Lymph Node Biopsy (SLNB)
Source: Int J Breast Cancer. 2026 Jun 26;2026:5039446. doi: 10.1155/ijbc/5039446 (PMC13305675; doi:10.1155/ijbc/5039446)
Supplement: Supplementary file 22 — Supporting Information 22 Figure S13 shows a comparison of the overall survival rate according to the presence of the PR hormone receptor. [file IJBC-2026-5039446-s005.docx]

| **Supplementary Table S13: Comparison of overall survival rate according to the presence of the PR hormone receptor (P = 0.05)** | | | | |
| --- | --- | --- | --- | --- |
| PR hormone receptor | Average | Standard deviation | 95 percent confidence interval | |
|  |  |  | Lower bound | Upper bound |
| Present | 16.798 | 0.612 | 15.598 | 17.999 |
| Unknown | 10.513 | 0.702 | 9.138 | 11.889 |
| Absent | 18.998 | 0.508 | 18.002 | 19.993 |
