# Supplementary material for: Comparing Disease‐Free Survival (DFS) and Overall Survival (OS) Rates in Breast Cancer Patients: Axillary Lymph Node Dissection (ALND) Versus Sentinel Lymph Node Biopsy (SLNB)
Source: Int J Breast Cancer. 2026 Jun 26;2026:5039446. doi: 10.1155/ijbc/5039446 (PMC13305675; doi:10.1155/ijbc/5039446)
Supplement: Supplementary file 24 — Supporting Information 24 Figure S14 shows a comparison of the disease‐free survival rate according to the presence of the PR hormone receptor. [file IJBC-2026-5039446-s007.docx]

| **Supplementary Table S14: Comparison of disease-free survival rate according to the presence of the PR hormone receptor (P = 0.001)** | | | | |
| --- | --- | --- | --- | --- |
| PR hormone receptor | Average | Standard deviation | 95 percent confidence interval | |
|  |  |  | Lower bound | Upper bound |
| Present | 16.255 | 0.666 | 14.949 | 17.560 |
| Unknown | 11.807 | 0.397 | 11.028 | 12.586 |
| Absent | 16.074 | 0.810 | 14.487 | 17.662 |
