# Supplementary material for: Comparing Disease‐Free Survival (DFS) and Overall Survival (OS) Rates in Breast Cancer Patients: Axillary Lymph Node Dissection (ALND) Versus Sentinel Lymph Node Biopsy (SLNB)
Source: Int J Breast Cancer. 2026 Jun 26;2026:5039446. doi: 10.1155/ijbc/5039446 (PMC13305675; doi:10.1155/ijbc/5039446)
Supplement: Supplementary file 25 — Supporting Information 25 Table S15 shows a comparison of the overall survival rate according to the presence of the P53 gene. [file IJBC-2026-5039446-s008.docx]

| **Supplementary Table S15: Comparison of overall survival rate according to the presence of P53 gene (P = 0.168)** | | | | |
| --- | --- | --- | --- | --- |
| P53 gene | Average | Standard deviation | 95 percent confidence interval | |
|  |  |  | Lower bound | Upper bound |
| Present | 15.738 | 1.024 | 13.731 | 17.746 |
| Unknown | 18.086 | 0.654 | 16.804 | 19.368 |
| Absent | 15.936 | 0.513 | 14.930 | 16.942 |
