# Supplementary material for: Comparing Disease‐Free Survival (DFS) and Overall Survival (OS) Rates in Breast Cancer Patients: Axillary Lymph Node Dissection (ALND) Versus Sentinel Lymph Node Biopsy (SLNB)
Source: Int J Breast Cancer. 2026 Jun 26;2026:5039446. doi: 10.1155/ijbc/5039446 (PMC13305675; doi:10.1155/ijbc/5039446)
Supplement: Supplementary file 27 — Supporting Information 27 Table S16 shows a comparison of the disease‐free survival rate according to the presence of the P53 gene. [file IJBC-2026-5039446-s010.docx]

| **Supplementary Table S16: Comparison of disease-free survival rate according to the presence of P53 gene (P = 0.846)** | | | | |
| --- | --- | --- | --- | --- |
| P53 gene | Average | Standard deviation | 95 percent confidence interval | |
|  |  |  | Lower bound | Upper bound |
| Present | 14.747 | 1.157 | 12.480 | 17.014 |
| Unknown | 17.103 | 0.537 | 16.051 | 18.155 |
| Absent | 14.851 | 0.639 | 13.598 | 16.104 |
