# Supplementary material for: Comparing Disease‐Free Survival (DFS) and Overall Survival (OS) Rates in Breast Cancer Patients: Axillary Lymph Node Dissection (ALND) Versus Sentinel Lymph Node Biopsy (SLNB)
Source: Int J Breast Cancer. 2026 Jun 26;2026:5039446. doi: 10.1155/ijbc/5039446 (PMC13305675; doi:10.1155/ijbc/5039446)
Supplement: Supplementary file 29 — Supporting Information 29 Table S17 shows a comparison of the overall survival rate according to the presence of the HER‐2 gene. [file IJBC-2026-5039446-s012.docx]

| **Supplementary Table S17: Comparison of overall survival rate according to the presence of HER-2 gene (P = 0.536)** | | | | |
| --- | --- | --- | --- | --- |
| HER-2 gene | Average | Standard deviation | 95 percent confidence interval | |
|  |  |  | Lower bound | Upper bound |
| Present | 17.131 | 1.243 | 14.694 | 19.567 |
| Unknown | 17.174 | 0.574 | 16.048 | 18.299 |
| Absent | 17.844 | 0.708 | 16.457 | 19.231 |
