# Supplementary material for: Comparing Disease‐Free Survival (DFS) and Overall Survival (OS) Rates in Breast Cancer Patients: Axillary Lymph Node Dissection (ALND) Versus Sentinel Lymph Node Biopsy (SLNB)
Source: Int J Breast Cancer. 2026 Jun 26;2026:5039446. doi: 10.1155/ijbc/5039446 (PMC13305675; doi:10.1155/ijbc/5039446)
Supplement: Supplementary file 31 — Supporting Information 31 Table S18 shows a comparison of the disease‐free survival rate according to the presence of the HER‐2 gene. [file IJBC-2026-5039446-s014.docx]

| **Supplementary Table S18: Comparison of disease-free survival rate according to the presence of HER-2 gene (P = 0.02)** | | | | |
| --- | --- | --- | --- | --- |
| HER-2 gene | Average | Standard deviation | 95 percent confidence interval | |
|  |  |  | Lower bound | Upper bound |
| Present | 13.086 | 0.633 | 11.844 | 14.327 |
| Unknown | 15.329 | 0.751 | 13.856 | 16.802 |
| Absent | 17.948 | 0.495 | 16.978 | 18.918 |
