# Supplementary material for: Comparing Disease‐Free Survival (DFS) and Overall Survival (OS) Rates in Breast Cancer Patients: Axillary Lymph Node Dissection (ALND) Versus Sentinel Lymph Node Biopsy (SLNB)
Source: Int J Breast Cancer. 2026 Jun 26;2026:5039446. doi: 10.1155/ijbc/5039446 (PMC13305675; doi:10.1155/ijbc/5039446)
Supplement: Supplementary file 33 — Supporting Information 33 Table S19 shows a comparison of the overall survival rate according to chemotherapy. [file IJBC-2026-5039446-s016.docx]

| **Supplementary Table S19: Comparison of overall survival rate according to chemotherapy (P = 0.002)** | | | | |
| --- | --- | --- | --- | --- |
| chemotherapy | Average | Standard deviation | 95 percent confidence interval | |
|  |  |  | Lower bound | Upper bound |
| Present | 17.980 | 0.547 | 16.907 | 19.053 |
| Neoadjuvant chemotherapy | 11.338 | 1.036 | 9.307 | 13.370 |
| Unknown | 16.667 | 1.392 | 13.948 | 19.406 |
| Absent | 12.288 | 0.096 | 12.101 | 12.476 |
