# Supplementary material for: Comparing Disease‐Free Survival (DFS) and Overall Survival (OS) Rates in Breast Cancer Patients: Axillary Lymph Node Dissection (ALND) Versus Sentinel Lymph Node Biopsy (SLNB)
Source: Int J Breast Cancer. 2026 Jun 26;2026:5039446. doi: 10.1155/ijbc/5039446 (PMC13305675; doi:10.1155/ijbc/5039446)
Supplement: Supplementary file 35 — Supporting Information 35 Table S20 shows a comparison of the disease‐free survival rate according to chemotherapy. [file IJBC-2026-5039446-s018.docx]

| **Supplementary Table S20: Comparison of disease-free survival rate according to chemotherapy (P = 0.294)** | | | | |
| --- | --- | --- | --- | --- |
| chemotherapy | Average | Standard deviation | 95 percent confidence interval | |
|  |  |  | Lower bound | Upper bound |
| Present | 16.998 | 0.557 | 15.906 | 19.090 |
| Neoadjuvant chemotherapy | 12.825 | 1.095 | 10.678 | 14.972 |
| Unknown | 12.096 | 0.610 | 10.901 | 13.291 |
| Absent | 11.603 | 0.254 | 11.104 | 12.101 |
