# Supplementary material for: Comparing Disease‐Free Survival (DFS) and Overall Survival (OS) Rates in Breast Cancer Patients: Axillary Lymph Node Dissection (ALND) Versus Sentinel Lymph Node Biopsy (SLNB)
Source: Int J Breast Cancer. 2026 Jun 26;2026:5039446. doi: 10.1155/ijbc/5039446 (PMC13305675; doi:10.1155/ijbc/5039446)
Supplement: Supplementary file 37 — Supporting Information 37 Table S21 shows a comparison of the overall survival rate according to hormone therapy. [file IJBC-2026-5039446-s020.docx]

| **Supplementary Table S21: Comparison of overall survival rate according to hormone therapy (P = 0.005)** | | | | |
| --- | --- | --- | --- | --- |
| hormone therapy | Average | Standard deviation | 95 percent confidence interval | |
|  |  |  | Lower bound | Upper bound |
| Present | 17.061 | 0.512 | 16.058 | 18.064 |
| Unknown | 8.284 | 1.028 | 6.269 | 10.298 |
| Absent | 18.578 | 0.771 | 17.067 | 20.089 |
