# Supplementary material for: Comparing Disease‐Free Survival (DFS) and Overall Survival (OS) Rates in Breast Cancer Patients: Axillary Lymph Node Dissection (ALND) Versus Sentinel Lymph Node Biopsy (SLNB)
Source: Int J Breast Cancer. 2026 Jun 26;2026:5039446. doi: 10.1155/ijbc/5039446 (PMC13305675; doi:10.1155/ijbc/5039446)
Supplement: Supplementary file 38 — Supporting Information 38 Figure S21 shows a comparison of the overall survival rate according to hormone therapy. [file IJBC-2026-5039446-s021.docx]

Survival Functions

Hormone therapy

Present Unknown Absent

censored- Present censored- Unknown censored- Absent


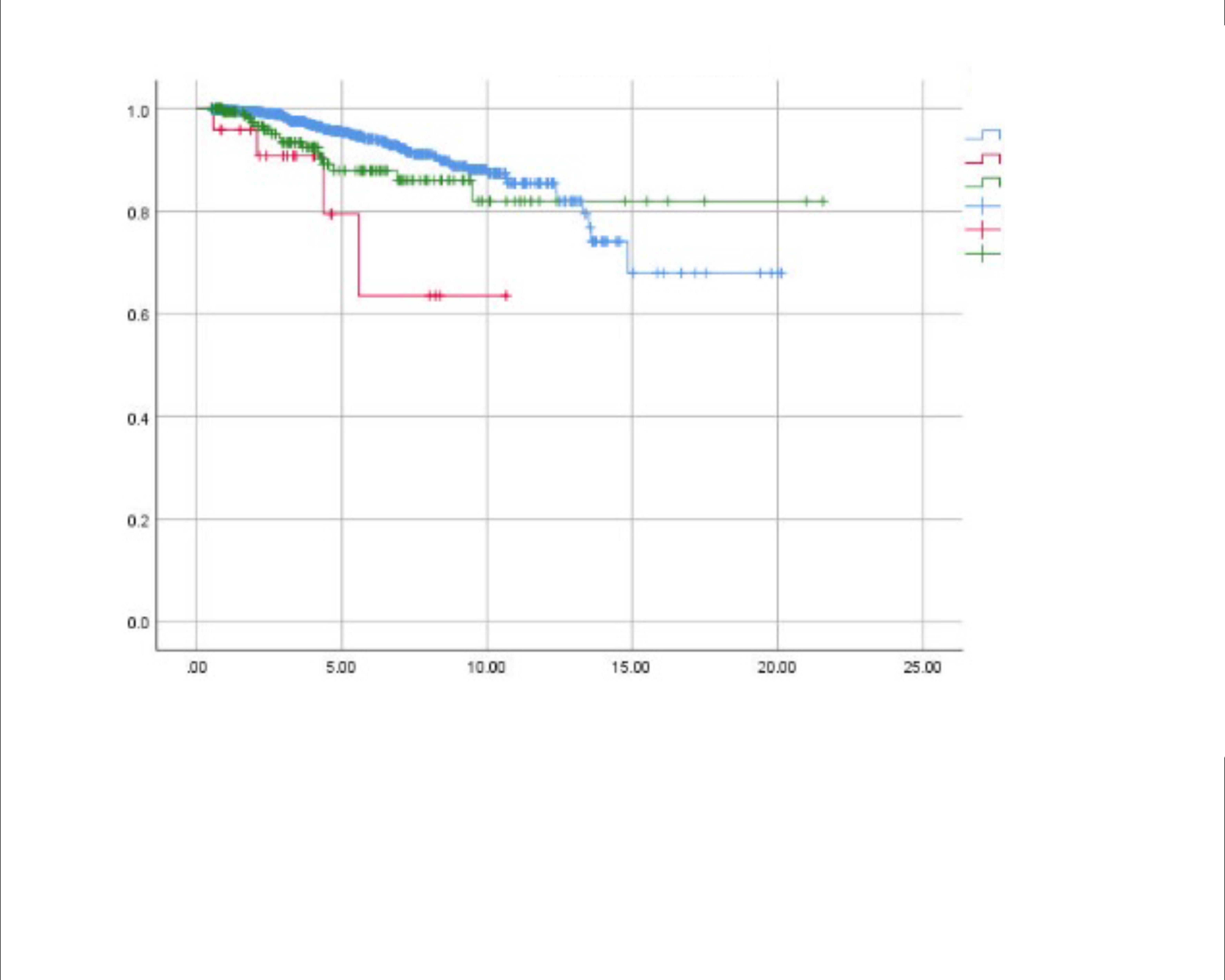


C u m S u r v i v a l

TIME.DEATH.YEAR

Supplementary Figure S21: Comparison of overall survival rate according to hormone therapy (P = 0.005)
