# Supplementary material for: Comparing Disease‐Free Survival (DFS) and Overall Survival (OS) Rates in Breast Cancer Patients: Axillary Lymph Node Dissection (ALND) Versus Sentinel Lymph Node Biopsy (SLNB)
Source: Int J Breast Cancer. 2026 Jun 26;2026:5039446. doi: 10.1155/ijbc/5039446 (PMC13305675; doi:10.1155/ijbc/5039446)
Supplement: Supplementary file 39 — Supporting Information 39 Table S22 shows a comparison of the disease‐free survival rate according to hormone therapy. [file IJBC-2026-5039446-s022.docx]

| **Supplementary Table S22: Comparison of disease-free survival rate according to hormone therapy (P = 0.044)** | | | | |
| --- | --- | --- | --- | --- |
| Hormone therapy | Average | Standard deviation | 95 percent confidence interval | |
|  |  |  | Lower bound | Upper bound |
| Present | 16.085 | 0.523 | 15.059 | 17.111 |
| Unknown | 9.692 | 0.647 | 8.424 | 10.959 |
| Absent | 17.084 | 0.824 | 15.469 | 18.698 |
