# Supplementary material for: Comparing Disease‐Free Survival (DFS) and Overall Survival (OS) Rates in Breast Cancer Patients: Axillary Lymph Node Dissection (ALND) Versus Sentinel Lymph Node Biopsy (SLNB)
Source: Int J Breast Cancer. 2026 Jun 26;2026:5039446. doi: 10.1155/ijbc/5039446 (PMC13305675; doi:10.1155/ijbc/5039446)
Supplement: Supplementary file 40 — Supporting Information 40 Figure S22 shows a comparison of the disease‐free survival rate according to hormone therapy. [file IJBC-2026-5039446-s023.docx]

Survival Functions

Hormone therapy


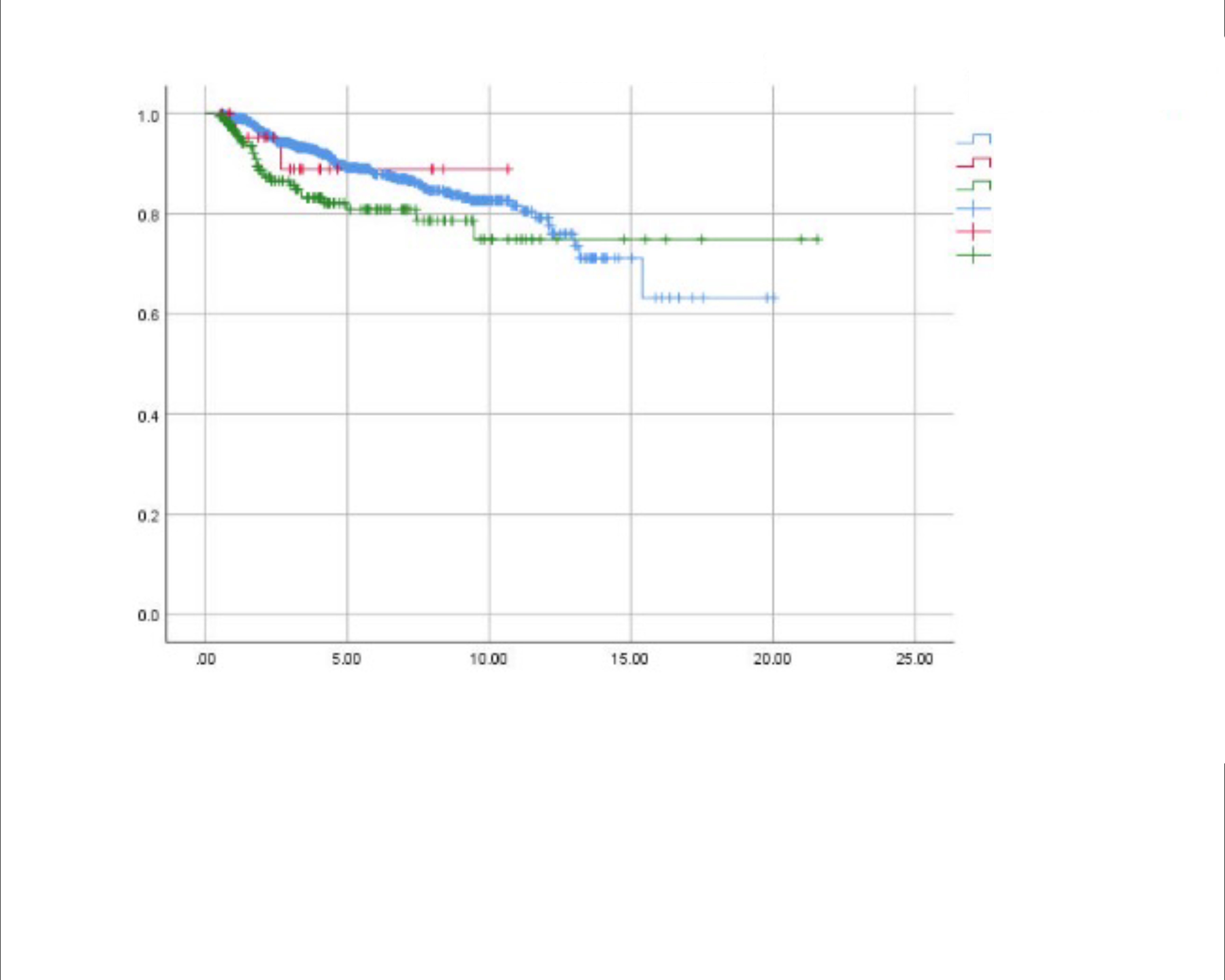


Present Unknown Absent

censored- Present censored- Unknown censored- Absent

C u m S u r v i v a l

TIME.REC.YEAR

Supplementary Figure S22: Comparison of disease-free survival rate according to hormone therapy (P = 0.044)
