# Supplementary material for: Comparing Disease‐Free Survival (DFS) and Overall Survival (OS) Rates in Breast Cancer Patients: Axillary Lymph Node Dissection (ALND) Versus Sentinel Lymph Node Biopsy (SLNB)
Source: Int J Breast Cancer. 2026 Jun 26;2026:5039446. doi: 10.1155/ijbc/5039446 (PMC13305675; doi:10.1155/ijbc/5039446)
Supplement: Supplementary file 41 — Supporting Information 41 Table S23 shows a comparison of the overall survival rate according to tumor size. [file IJBC-2026-5039446-s024.docx]

| **Supplementary Table S23: Comparison of overall survival rate according to tumor size (P≤0.001)** | | | | |
| --- | --- | --- | --- | --- |
| Tumor size | Average | Standard deviation | 95 percent confidence interval | |
|  |  |  | Lower bound | Upper bound |
| Less than 2 cm | 19.887 | 0.641 | 18.630 | 21.144 |
| Between 2 and 5 cm | 17.811 | 0.584 | 16.666 | 18.957 |
| More than 5 cm | 13.360 | 1.187 | 11.033 | 15.688 |
| Unknown | 14.681 | 0.766 | 13.179 | 16.182 |
