# Supplementary material for: Comparing Disease‐Free Survival (DFS) and Overall Survival (OS) Rates in Breast Cancer Patients: Axillary Lymph Node Dissection (ALND) Versus Sentinel Lymph Node Biopsy (SLNB)
Source: Int J Breast Cancer. 2026 Jun 26;2026:5039446. doi: 10.1155/ijbc/5039446 (PMC13305675; doi:10.1155/ijbc/5039446)
Supplement: Supplementary file 42 — Supporting Information 42 Figure S23 shows a comparison of the overall survival rate according to tumor size. [file IJBC-2026-5039446-s025.docx]

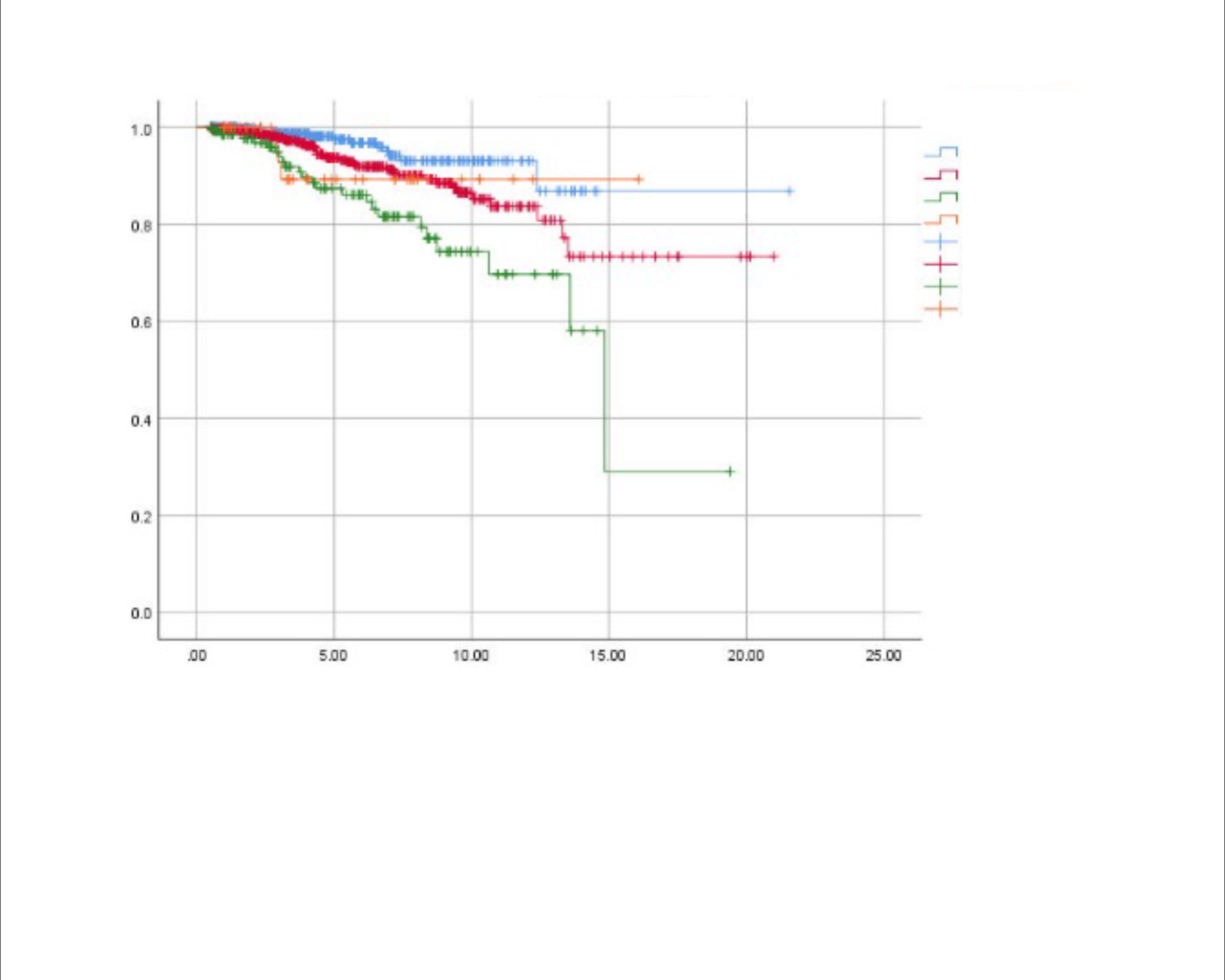
Survival Functions Tumor size

C u m S u r v i v a l

less than 2 cm between 2 and 5 cm more than 5 cm Censored- Unknown

Censored- less than 2 cm Censored- between 2 and 5 cm Censored- more than 5 cm Censored- Unknown

TIME.DEATH.YEAR

Supplementary Figure S23: Comparison of overall survival rate according to tumor size (P≤0.001)
