# Supplementary material for: Comparing Disease‐Free Survival (DFS) and Overall Survival (OS) Rates in Breast Cancer Patients: Axillary Lymph Node Dissection (ALND) Versus Sentinel Lymph Node Biopsy (SLNB)
Source: Int J Breast Cancer. 2026 Jun 26;2026:5039446. doi: 10.1155/ijbc/5039446 (PMC13305675; doi:10.1155/ijbc/5039446)
Supplement: Supplementary file 43 — Supporting Information 43 Table S24 shows a comparison of the disease‐free survival rate according to tumor size. [file IJBC-2026-5039446-s026.docx]

| **Supplementary Table S24: Comparison of disease-free survival rate according to tumor size (P = 0.018)** | | | | |
| --- | --- | --- | --- | --- |
| Tumor size | Average | Standard deviation | 95 percent confidence interval | |
|  |  |  | Lower bound | Upper bound |
| Less than 2 cm | 18.255 | 0.718 | 16.848 | 19.663 |
| Between 2 and 5 cm | 16.857 | 0.644 | 15.595 | 18.119 |
| More than 5 cm | 11.986 | 0.716 | 10.582 | 13.390 |
| Unknown | 14.862 | 0.680 | 13.528 | 16.195 |
