# Supplementary material for: Comparing Disease‐Free Survival (DFS) and Overall Survival (OS) Rates in Breast Cancer Patients: Axillary Lymph Node Dissection (ALND) Versus Sentinel Lymph Node Biopsy (SLNB)
Source: Int J Breast Cancer. 2026 Jun 26;2026:5039446. doi: 10.1155/ijbc/5039446 (PMC13305675; doi:10.1155/ijbc/5039446)
Supplement: Supplementary file 45 — Supporting Information 45 Table S25 shows a comparison of the overall survival rate according to the stage of the disease. [file IJBC-2026-5039446-s028.docx]

| **Supplementary Table S25: Comparison of overall survival rate according to the stage of the disease (P≤0.001)** | | | | |
| --- | --- | --- | --- | --- |
| Stage of the disease | Average | Standard deviation | 95 percent confidence interval | |
|  |  |  | Lower bound | Upper bound |
| Stage0 | 11.586 | 0.632 | 10.347 | 12.825 |
| Stage1 | 19.634 | 0.971 | 17.730 | 21.537 |
| Stage2 | 18.166 | 0.573 | 17.043 | 19.289 |
| Stage3 | 12.282 | 0.560 | 11.184 | 13.381 |
| Stage4 | 7.673 | 1.559 | 4.618 | 10.728 |
| unknown | 14.210 | 1.736 | 10.808 | 17.612 |
