# Supplementary material for: Comparing Disease‐Free Survival (DFS) and Overall Survival (OS) Rates in Breast Cancer Patients: Axillary Lymph Node Dissection (ALND) Versus Sentinel Lymph Node Biopsy (SLNB)
Source: Int J Breast Cancer. 2026 Jun 26;2026:5039446. doi: 10.1155/ijbc/5039446 (PMC13305675; doi:10.1155/ijbc/5039446)
Supplement: Supplementary file 46 — Supporting Information 46 Figure S25 shows a comparison of the overall survival rate according to the stage of the disease. [file IJBC-2026-5039446-s029.docx]

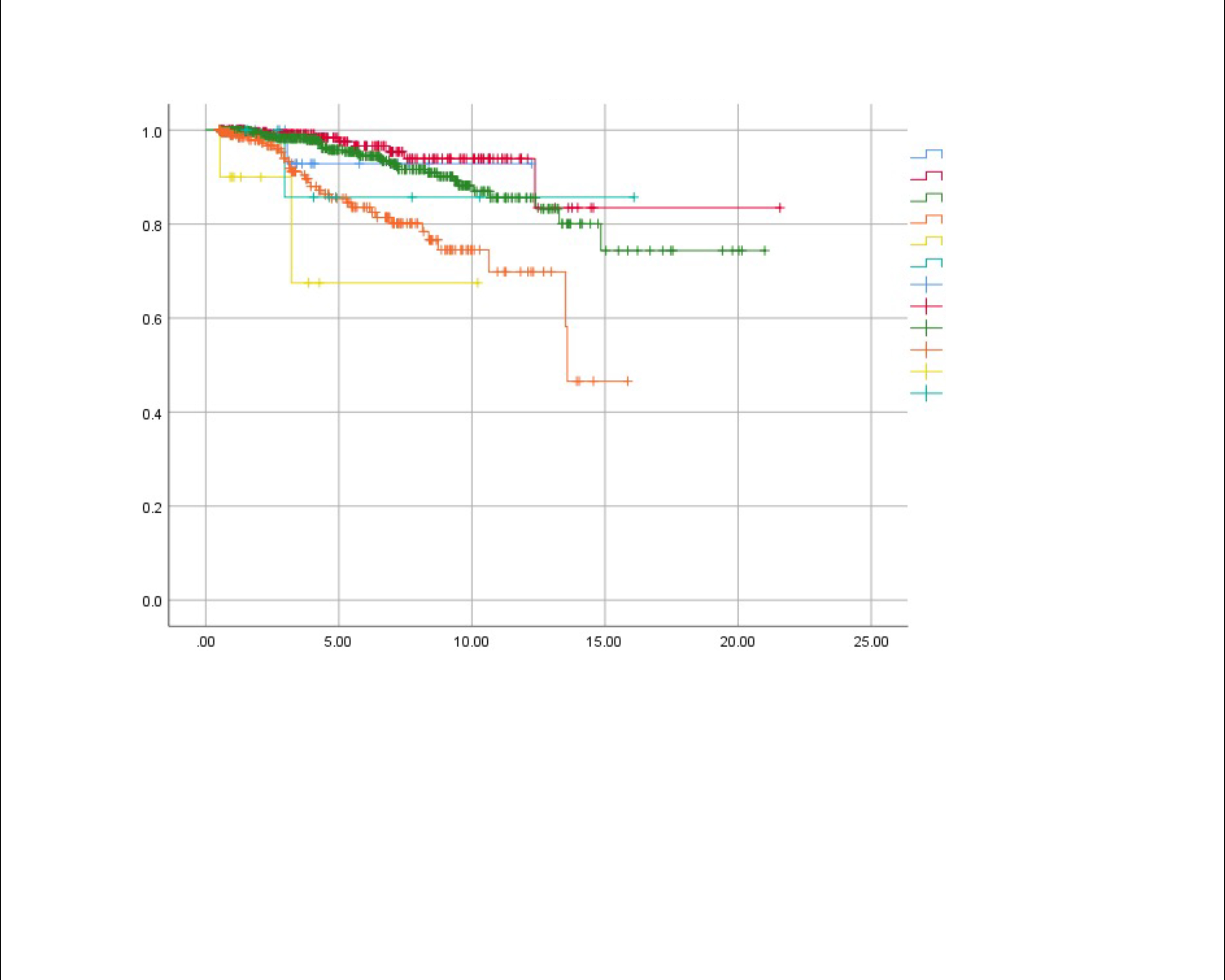
Survival Functions Stage

C u m S u r v i v a l

stage 0 stage 1 stage 2 stage 3 stage 4 unknown

stage 0-censored stage 1-censored stage 2-censored stage 3-censored stage 4-censored unknown-censored

TIME.DEATH.YEAR

Supplementary Figure S25: Comparison of overall survival rate according to the stage of the disease (P≤0.001)
