# Supplementary material for: Comparing Disease‐Free Survival (DFS) and Overall Survival (OS) Rates in Breast Cancer Patients: Axillary Lymph Node Dissection (ALND) Versus Sentinel Lymph Node Biopsy (SLNB)
Source: Int J Breast Cancer. 2026 Jun 26;2026:5039446. doi: 10.1155/ijbc/5039446 (PMC13305675; doi:10.1155/ijbc/5039446)
Supplement: Supplementary file 48 — Supporting Information 48 Figure S26 shows a comparison of the disease‐free survival rate according to the stage of the disease. [file IJBC-2026-5039446-s031.docx]

# Survival Functions

Stage


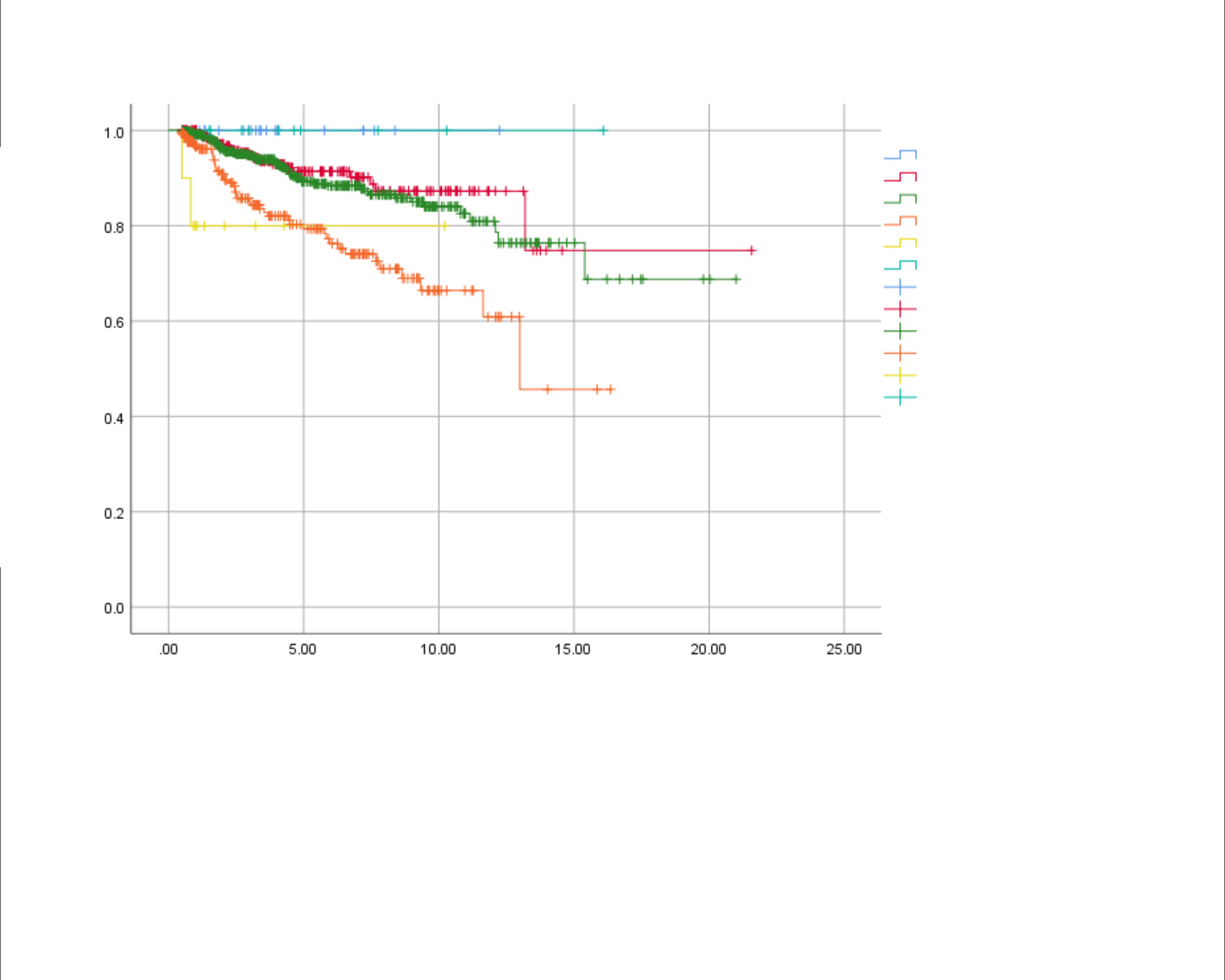


C u m S u r v i v a l

stage 0 stage 1 stage 2 stage 3 stage 4 unknown

stage 0-censored stage 1-censored stage 2-censored stage 3-censored stage 4-censored unknown-censored

# TIME.REC.YEAR

Supplementary Figure S26: Comparison of disease-free survival rate according to the stage of the disease (P≤0.001)
