# Supplementary material for: Comparing Disease‐Free Survival (DFS) and Overall Survival (OS) Rates in Breast Cancer Patients: Axillary Lymph Node Dissection (ALND) Versus Sentinel Lymph Node Biopsy (SLNB)
Source: Int J Breast Cancer. 2026 Jun 26;2026:5039446. doi: 10.1155/ijbc/5039446 (PMC13305675; doi:10.1155/ijbc/5039446)
Supplement: Supplementary file 49 — Supporting Information 49 Table S27 shows a comparison of the overall survival rate according to pathology. [file IJBC-2026-5039446-s032.docx]

| **Supplementary Table S27: Comparison of overall survival rate according to pathology (P = 0.025)** | | | | |
| --- | --- | --- | --- | --- |
| Pathology | Average | Standard deviation | 95 percent confidence interval | |
|  |  |  | Lower bound | Upper bound |
| DCIS | 17.711 | 0.561 | 16.612 | 18.810 |
| OTHER | 18.732 | 0.393 | 17.962 | 19.501 |
