# Supplementary material for: Comparing Disease‐Free Survival (DFS) and Overall Survival (OS) Rates in Breast Cancer Patients: Axillary Lymph Node Dissection (ALND) Versus Sentinel Lymph Node Biopsy (SLNB)
Source: Int J Breast Cancer. 2026 Jun 26;2026:5039446. doi: 10.1155/ijbc/5039446 (PMC13305675; doi:10.1155/ijbc/5039446)
Supplement: Supplementary file 51 — Supporting Information 51 Table S28 shows a comparison of the disease‐free survival rate according to tumor size. [file IJBC-2026-5039446-s034.docx]

| **Supplementary Table S28: Comparison of disease-free survival rate according to tumor size (P = 0.893)** | | | | |
| --- | --- | --- | --- | --- |
| Tumor size | Average | Standard deviation | 95 percent confidence interval | |
|  |  |  | Lower bound | Upper bound |
| DCIS | 16.905 | 0.582 | 15.765 | 18.046 |
| OTHER | 17.080 | 0.504 | 16.092 | 18.069 |
